# Quantitative Proteomics and CRISPR/Cas9 Editing Reveal UPR‐Mediated Control of Immunoglobulin Homeostasis in Hybridomas

**DOI:** 10.1002/advs.202514140

**Published:** 2026-01-20

**Authors:** Rubing Zou, Xinying Lu, Ying Liu, Peiyu Yang, Shuo Sun, Yihua Liu, Yinyuan Mo, Guonian Zhu, Jae Seong Lee, Yirong Guo

**Affiliations:** ^1^ Institute of Pesticide and Environmental Toxicology Zhejiang Key Laboratory of Biology and Ecological Regulation of Crop Pathogens and Insects Ministry of Agriculture and Rural Affairs Key Laboratory of Molecular Biology of Crop Pathogens and Insect Pests Zhejiang University Hangzhou China; ^2^ School of Life Science Jining Medical University Rizhao China; ^3^ Research Institute of Subtropical Forestry Chinese Academy of Forestry Hangzhou China; ^4^ Institute of Clinical Medicine Zhejiang Provincial People's Hospital of Hangzhou Medical College Hangzhou China; ^5^ Graduate School of Engineering Biology KAIST Daejeon Republic of Korea; ^6^ KI for the BioCentury KAIST Daejeon Republic of Korea

**Keywords:** antibody production, CRISPR/Cas9, hybridoma, immunoglobulin, proteomics

## Abstract

Despite their tremendous economic value, monoclonal antibodies are often compromised by the loss of immunoglobulin (Ig) chains, which disrupts antibody homeostasis and quality control. Through subclone screening and characterization, we identified that the loss of Ig production impaired the recognition ability of hapten‐specific hybridomas. Proteomic analysis further highlighted the critical role of the unfolded protein response (UPR) pathway in regulating aberrant Ig chain production. Using CRISPR/Cas9‐mediated knockout and rescue experiments, we revealed the importance of the UPR pathway in facilitating hybridoma antibody production by targeting *Xbp1*s, an active transcription factor downstream of UPR signaling. With CRISPR/HDR, we inserted a fluorescent mGFP tag into the endogenous *Hspa5* gene (encoding BiP, the master regulator of the UPR pathway), enabling in situ and real‐time monitoring of UPR activation. A strong negative correlation (R^2^ = 0.86) was observed between intracellular mGFP signals and IgG levels in the engineered system, indicating a close relationship between UPR activation and Ig production. Fluorescence‐activated cell sorting of high‐mGFP populations identified two dysfunctional subclones that failed to secret Ig, validating the system’s effectiveness in tracing Ig homeostasis. In summary, this study provides new insights into UPR‐mediated regulation of Ig synthesis and offers a novel UPR‐based reporter system for monitoring antibody stability.

## Graphical abstract

BCR sequencing and subclone analysis correlated immunoglobulin (Ig) chain loss in dysfunctional hybridomas with disrupted monoclonal antibody homeostasis. Proteomics‐guided CRISPR/Cas9 editing revealed that the unfolded protein response (UPR) regulates aberrant Ig synthesis. A novel UPR‐based fluorescent reporter system was further established via CRISPR/HDR, enabling real‐time monitoring of UPR activation and antibody stability in hybridomas.

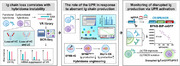

## 1 Introduction

Since its development in 1975, the hybridoma technique has become one of the most commonly used methods for monoclonal antibody discovery and production. Numerous hybridomas have been developed and widely applied in pharmaceuticals, clinical diagnostics and pollutant monitoring, creating a billion‐dollar market and playing a critical role in safeguarding human health [1, 2]. By enabling in vitro culturing, fed‐batch and bioreactor cultures of antigen‐specific hybridomas provide animal‐friendly approaches for large‐scale antibody production. However, achieving high‐quality and well‐controlled antibody production in hybridomas remains challenging, as the frequent loss of immunoglobulin (Ig) chains during long‐term cultivation has been reported across laboratories worldwide since 1980 [3, 4, 5]. This bottleneck disrupts antibody yield and increases the workload required for functional hybridoma screening, making industrial antibody discovery time‐consuming and labor‐intensive. Moreover, the loss of Ig chains hinders the identification of naturally folded Ig genes in hybridomas, thereby affecting the expression of recombinant products [6, 7], as well as hybridoma‐based antibody engineering studies [8, 9]. Therefore, there is a critical need to develop an efficient and effective approach to monitor hybridoma homeostasis and track Ig production.

Various fluorescence reporter systems have been developed for dynamic monitoring of cellular retox homeostasis [10], lipid droplets and lysosomes [11], but endogenous systems for in situ analysis of Ig are largely lacking. Traditional end‐point analytical methods, such as enzyme‐linked immunosorbent assays (ELISAs), offer robust approaches for analyzing Ig levels and the specificity of hapten‐specific hybridomas. However, real‐time and in situ monitoring of antibody secretion in living cells remains challenging, as Ig levels are often influenced by cell density and proliferation status. Fluorescence‐activated cell sorting (FACS) has been employed to identify and isolate antigen‐specific antibody‐secreting cells, but conventional staining of cell surface Ig is often unpredictable, and biased screening may occur due to non‐specific binding of fluorescent probes or inappropriate staining procedures [12, 13, 14]. Additionally, growing evidence indicates substaintial genetic heterogeneity among hybridoma subclones, which arises from multiple factors such as point mutations [15, 16, 17] and chromosomal rearrangements [3, 18]. The emergence of aberrant functional or non‐functional Ig chains alters the transcriptional profiles of variable heavy chain (VH) and variable light chain (VL) genes, creating bottlenecks for the identification of hapten‐specific Ig genes and the analysis of their transcriptional expression levels [17]. Given its ability to profile protein expression and map molecular interactions, high‐throughput proteomics has been widely applied in exposome analysis [19, 20] and diagnostic marker discovery [21, 22], providing valuable insights into dynamic multi‐omics regulation and system‐wide biological changes. Herein, we propose that identifying key regulators mediating Ig chain loss could provide an effective strategy to monitor hybridoma homeostasis.

The endoplasmic reticulum (ER) and Golgi apparatus are important organelles that facilitate protein folding, assembly and maturation. However, the high demand and rapid synthesis of secretory cargo impose intensive stress on this system. In response to such endogenous conditions, eukaryotic cells activate the unfolded protein response (UPR), an evolutionarily conserved signaling cascade that mitigates ER stress and restores cellular homeostasis by regulating protein expression, cell proliferation and apoptosis. The UPR pathway, driven by the transcriptional activator *Xbp1*, supports antibody production by expanding ER size and enhancing protein‐folding capability in plasma cells (long‐lived antibody‐producing cells) [23, 24]. Moreover, UPR activation has been associated with increased antibody productivity [25] and reduced cell growth in mammalian cells (e.g., Chinese hamster ovary, CHO) [26]. Although previous studies have revealed that chromosome loss [3], epigenetic silencing [27] and RNA post‐transcriptional modification [28] are major factors influencing Ig homeostasis, the mechanisms underlying the regulation of hybridoma homeostasis control remain poorly understood. Given its essential role in protein quality control, whether the UPR pathway is also crucial for maintaining hybridoma homeostasis, particularly in the context of Ig chain loss, still lacks supporting evidence. A recent study has hypothesized that misfolded heavy chains (HCs) may drive ER stress and cause homeostatic failure, potentially explaining the toxicity of free HCs in hybridomas. However, this hypothesis still lacks experimental validation [3, 29].

In this study, multiple functional and naturally HC‐deficient dysfunctional subclones were isolated through limiting dilution screening. A previously developed hapten‐specific hybridoma cell line (clone number: BJQ‐FS1), which secrets monoclonal antibodies against chlorothalonil (a representative fungicide), was selected as the model. The correlation between Ig chain loss and hybridoma homeostasis disruption was investigated via phenotypic characterization and high‐throughput B‐cell receptor repertoire sequencing (BCR‐Seq). Integrating proteomic analysis, the role of the UPR pathway in regulating hybridoma Ig production was further examined through CRISPR/Cas9‐mediated knockout and rescue experiments. To enable real‐time and in situ monitoring of disrupted Ig production, we established a novel hybridoma fluorescent reporter system by inserting an endogenous mGFP tag into the locus of BiP (a master activator of the UPR pathway) using CRISPR/HDR. Finally, the feasibility of this intracellular reporter system was validated by assessing the correlation between mGFP intensity and Ig expression levels, providing an effective tool for monitoring hybridoma homeostasis and its antibody production.

## 2 Results

### 2.1 Loss of Ig Chains Impairs Specific Recognition of Hybridomas

**FIGURE 1:**
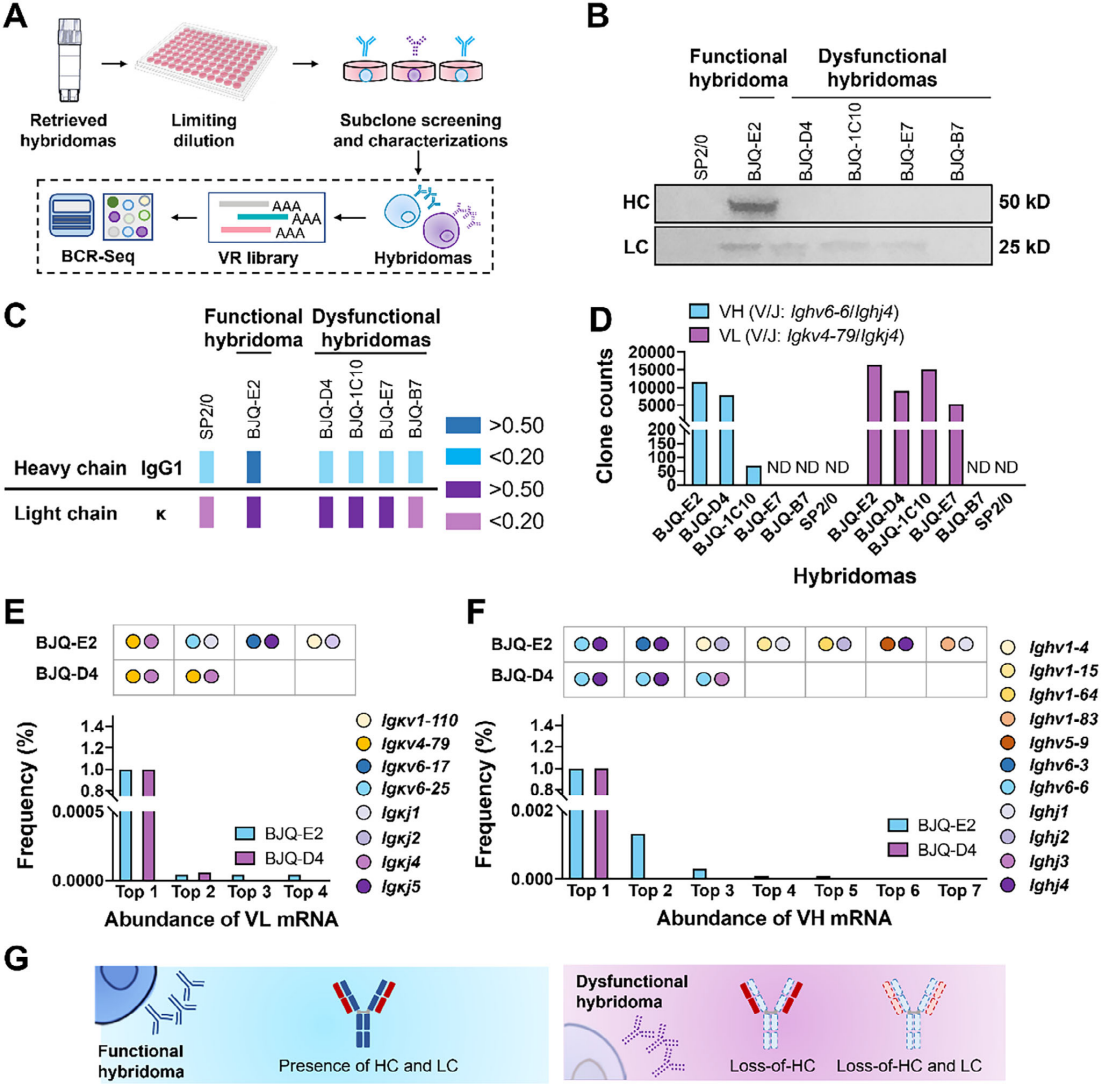
Correlation between Ig chain loss and homeostasis disruption in hybridomas. (A) Schematic illustration. The VR mRNA was profiled via B‐cell receptor repertoire sequencing (BCR‐Seq), followed by the isolation of target subclones through limiting dilution and characterization. Hapten‐specific BJQ‐FS1 and the myeloma SP2/0 (fusion partner) were selected for investigation. (B) Western blot analysis of heavy chain (HC) and light chain (LC) expression in five different BJQ‐derived hybridomas (BJQ‐E2/D4/1C10/E7/B7) and myeloma SP2/0. (C) Isotype determination. Hybridoma supernatants were harvested and analyzed using an HRP‐labeled goat‐anti‐mouse HC (IgG1/G2a/G2b/G3/M/A) and LC (κ/λ) ELISA kit (n = 3). Dark blue and dark purple indicate OD_450nm_ > 0.50 for HC and LC, respectively. Light blue and light purple represent average OD_450nm_ < 0.20 for HC and LC, respectively. (D) Clone counts of active VR mRNA in hybridomas. (E,F) Determined frequency of the top VL mRNA (E) and VH mRNA (F) in BJQ‐E2 and BJQ‐D4. Colored dots represent different V/J germline genes. (G) Expression of Ig chains in functional and dysfunctional hybridomas. The functional hybridoma (BJQ‐E2) expresses both HC and LC (HC^+^/LC^+^). Naturally occurring HC loss (HC^−^/LC^+^: BJQ‐D4/1C10/E7), as well as the simultaneous loss of both HC and LC (HC^−^/LC^−^: BJQ‐B7), are characteristic features of dysfunctional hybridomas.

Subclones of the hapten‐specific hybridoma BJQ‐FS1 (a cell line secrets IgG against chlorothalonil) were screened via limiting dilution in this study (Figure 1A). In non‐competitive ELISA, the supernatants of BJQ‐D4/1C10/E7/B7 (OD_450nm_: 0.05–0.09) and the myeloma SP2/0 (OD_450nm_: 0.04) exhibited significantly decreased signals, compared to BJQ‐E2 (OD_450nm_: 2.69). Meanwhile, the former hybridomas exhibited dramatically decreased level of IgG proteins than their functional sibling (Figure S1). Western blot analysis further revealed the disappearance of the HC (50 kD) and the presence of the LC (25 kD) in BJQ‐D4/1C10/E7. Neither HC nor LC was found in BJQ‐B7 and SP2/0 (Figure 1B). Isotype determination confirmed the presence of IgG1 and κ chains in the functional BJQ‐E2, while the dysfunctional hybridomas (BJQ‐D4/1C10/E7) showed loss of the IgG1 chain but retained κ chain production. Neither IgG1 nor κ chains were found in BJQ‐B7 or the parental myeloma cell line SP2/0 (Figure 1C). The above results indicated that the loss of Ig chains is associated with impaired antigen specificity in hybridomas.

To investigate the cause of Ig chain loss in dysfunctional hybridomas, BCR‐Seq analysis was conducted to trace the transcription patterns of VRs by generating VH and VL libraries from five BJQ‐derived hybridomas (Figure 1D). First, VH (Figure S2) and VL (Figure S3) sequences of BJQ‐E2 were obtained through V‐Region Sanger sequencing, with V/J classification of *Ighv6‐6*/*Ighj4* and *Igκv4‐79*/*Igκj4*, respectively (Table S1). Accordingly, the expressed full‐length IgG recombinant antibody (IC_50_: 0.51 ng/mL) exhibited a sensitivity similar to that of the ascites‐derived monoclonal antibody (IC_50_: 0.87 ng/mL) in indirect competitive ELISA, confirming the accuracy and reliability of the sequenced VR. Furthermore, the expression abundances of the identified VRs in different BJQ‐derived subclones were analyzed using BCR‐Seq. Target VH (clone counts: 11,619) and VL (clone counts: 16,350) with the same V/J classification were successfully traced in BJQ‐E2 (Figure 1D). Meanwhile, active VL (clone counts: 5,427∼15,142) was also found in BJQ‐D4/1C10/E7 (HC^−^/LC^+^), but failed to be detected in BJQ‐B7 (HC^−^/LC^−^) and SP2/0. Despite the lack of HC, target VH with significantly reduced abundance was also identified in BJQ‐D4 (clone counts: 7,956) and BJQ‐1C10 (clone counts: 70), but not in BJQ‐B7 and BJQ‐E7. Moreover, BJQ‐E2 and BJQ‐D4 displayed varied V/J pairs with different frequencies and distinct CDR3 regions, indicating the common presence of multiple aberrant VH (Table S2) and VL (Table S3). The identified active VH and VL were found in both BJQ‐E2 and BJQ‐D4 with the highest abundance (99.9%), indicating the effectiveness of BCR‐Seq in identifying hapten‐specific VR mRNA in hybridomas (Figure 1E,F). Together, these results reveal the correlation between the loss of Ig chains and the emergence of dysfunctional hybridomas, highlighting the importance of monitoring Ig chain production in hybridomas (Figure 1G).

### 2.2 The UPR Pathway Played an Important Role in Regulating Hybridoma Homeostasis for Ig Production

**FIGURE 2:**
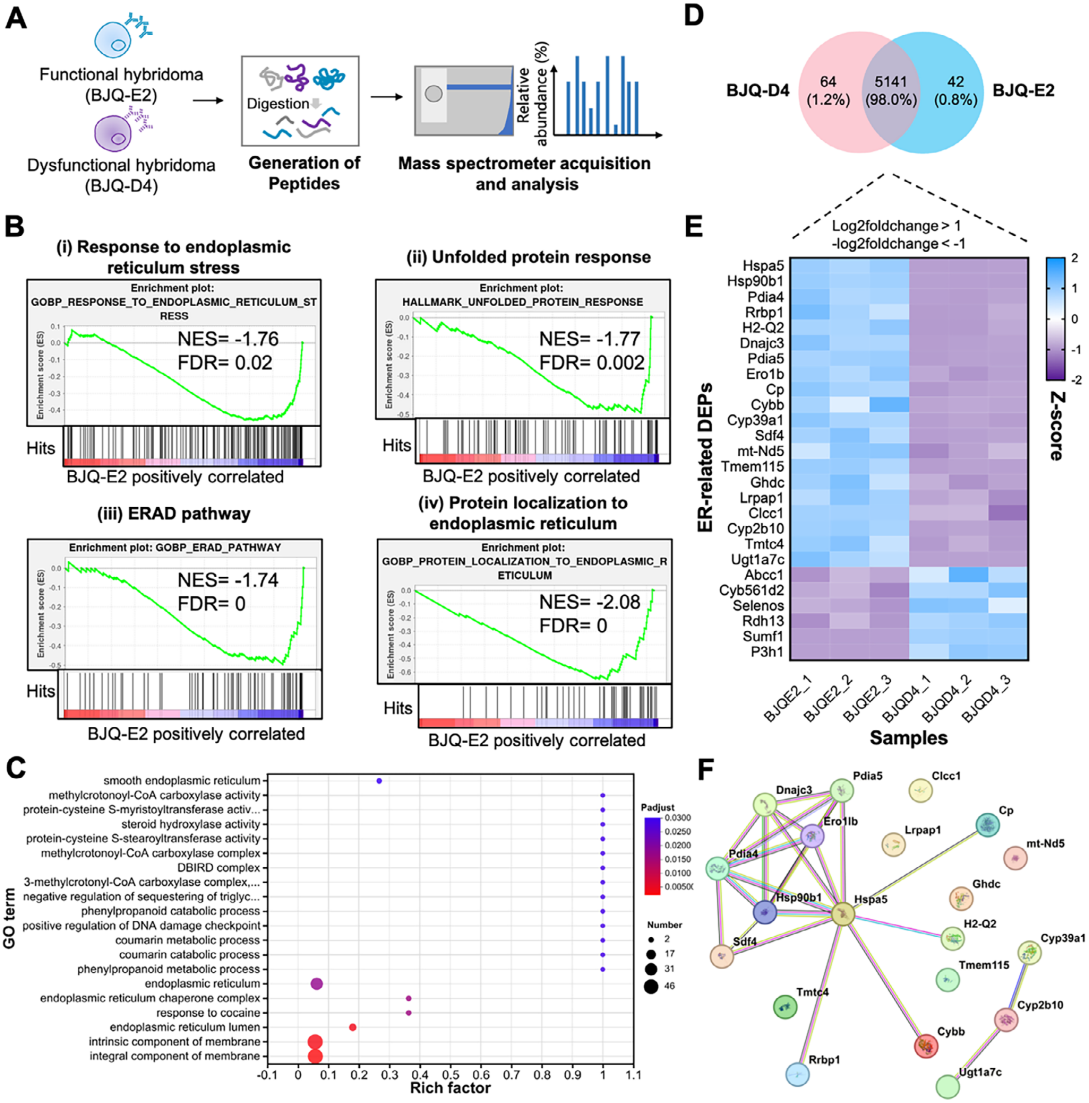
Identification of important pathway and genes in response to hybridoma Ig‐chain loss via high‐throughput proteomics. (A) Schematic illustruation of quantitative proteomics of hybridomas. Functional BJQ‐E2 and dysfunctional BJQ‐D4 were selected for generating peptides and then used for LC‐MS/MS analysis. Three biological replicates were used. The bioinformatic analysis was conducted by selecting BJQ‐E2 as the control (BJQ‐D4 vs BJQ‐E2). (B) Selected pathways identified by GSEA analysis. (i) Response to endoplasmic reticulum stress. (ii) Unfolded protein response. (iii) ERAD pathway. (iv) Protein localization to endoplasmic reticulum. (C) Enriched Top 20 GO terms using the down‐regulated DEPs in dysfunctional BJQ‐D4 (p < 0.05). (D) Venn diagram of protein expression in BJQ‐E2 and BJQ‐D4. In total 5,141 proteins were expressed in both hybridomas. (E) The heatmap of the significantly up‐regulated (n = 6) and down‐regulated ER‐related DEPs (n = 20). The DEPs were identified using a criterion of |log2foldchange| > 1. (F) The protein‐protein interaction (PPI) of the down‐regulated ER‐related DEPs.

Theoretically, the existence of non‐functional transcripts and the disappearance of Ig mRNA may disrupt IgG assembly, which could affect the function of other cellular components and ultimately compromise hybridoma homeostasis. Here, we employed quantitative proteomics to analyze how hybridomas respond to disordered Ig chain production, using the functional BJQ‐E2 (HC^+^/LC^+^) and dysfunctional BJQ‐D4 (HC^−^/LC^+^) subclones as models for sequencing, since they share relatively similar VH mRNA abundance but differ in HC expression (Figure 2A). Based on protein subcellular localization, the identified proteins (total number: 5,247) were distributed in the cytoplasm (53.8%), nuclear (21.7%), mitochondrial (11.2%), ER (4.3%) and Golgi apparatus (3.0%), respectively (Figure S4A,B). Of note, 12.6% and 11.5% of the identified proteins from the Golgi apparatus and ER were classified as DEPs, which was higher than in the cytoplasm (6.2%), nuclear (4.5%) and mitochondrial (6.8%) (Figure S4C,D). Moreover, GSEA further revealed that gene sets related to “response to endoplasmic reticulum stress” (NES: ‐1.76, FDR: 0.02) (Figure 2B‐i), “unfolded protein response” (UPR) (NES: −1.77, FDR: 0.002) (Figure 2B‐ii), “ERAD pathways” (NES: ‐1.74, FDR: 0) (Figure 2B‐iii) and “Protein localization to endoplasmic reticulum” (NES: −2.08, FDR: 0) (Figure 2B‐iv) were significantly enriched in functional BJQ‐E2. Additionally, GO terms related to the “endoplasmic reticulum lumen”, “endoplasmic reticulum chaperone complex”, “endoplasmic reticulum” and “smooth endoplasmic reticulum” were significantly enriched based on the analysis of down‐regulated DEPs in dysfunctional BJQ‐D4 compared to functional BJQ‐E2 (Figure 2C). By staining hybridomas with ER‐Tracker Red, we observed that functional hybridoma exhibited a significantly higher fluorescence intensity than its HC‐deficient subclone (Figure S5A). Similarly, BFNB‐derived hybridomas (BFNB‐4E: secreting hapten‐specific antibodies against carbofuran; BFNB‐11F: HC‐deficient dysfunctional sibling) showed the same trend (Figure S5B). These results indicated that the ER and the UPR pathway play important roles in facilitating antibody production and maintaining hybridoma homeostasis. As shown in the Venn diagram, 64 and 42 proteins were specifically expressed in BJQ‐D4 and BJQ‐E2, respectively (Figure 2D). Accordingly, 26 ER‐related DEPs were found, with 20 DEPs significantly down‐regulated in dysfunctional BJQ‐D4 (Figure 2E). As depicted, *Hspa5* was identified as the core protein in the PPI network, indicating its critical role in regulating homeostasis and antibody production in hybridomas (Figure 2F).

### 2.3 *Xbp1s* Positively Regulate IgG Expression in Hybridomas

**FIGURE 3:**
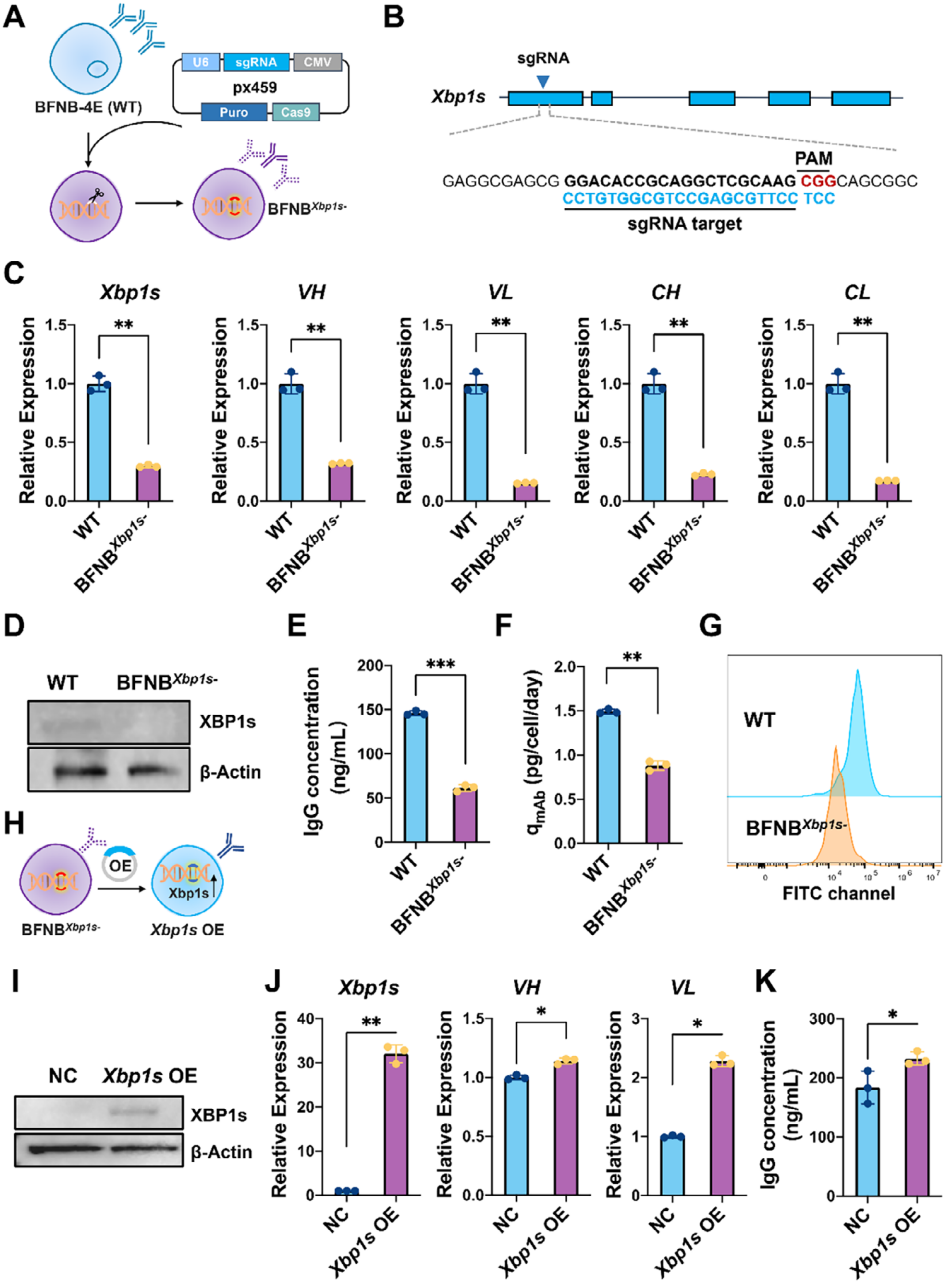
Investigation of the role of *Xbp1s* in regulating hybridoma antibody production through CRISPR/Cas9 knockout and rescue experiments. (A) Schematic illustration of CRISPR/Cas9‐mediated *Xbp1s* knockout in hybridomas. The hapten‐specific hybridoma BFNB‐4E (wild type, WT) was used for gene editing. The activator gene of the UPR pathway, *Xbp1s*, was selected as the target. (B) An sgRNA was designed to target exon 1 of *Xbp1s*. (C) The mRNA expression levels of *Xbp1s* and antibody genes (*VH*, *VL*, *CH* and *CL*) in WT hybridoma and the edited hybridoma BFNB*^Xbp1s−^*. (D) Western blot analysis of XBP1s expression in WT and BFNB*^Xbp1s−^*. Total proteins were extracted after exposure to tunicamycin (TM) at 2 mg/L for 8 h. (E‐G) Quantification of secreted IgG levels (E), q_mab_ (F) and the membrane‐located IgG level (G) in WT and BFNB*^Xbp1s−^*. (H) Schematic illustration of a rescue assay. The *Xbp1s* OE cell line was developed by restoring XBP1s expression in BFNB*^Xbp1s−^* using an *Xbp1s* overexpression (OE) plasmid. (I) Western blot analysis of XBP1s expression in BFNB*^Xbp1s−^* (negative control, NC) and X*bp1s* OE. (J) The mRNA expression levels of *Xbp1s* and antibody genes (*VH* and *VL*) in BFNB*^Xbp1s−^* (NC) and X*bp1s* OE. (K) Secreted IgG levels in BFNB *^Xbp1s^* (NC) and X*bp1s* OE. Three biological replicates were performed, and error bars represent the standard deviation. Statistical significance was analyzed using Student's *t*‐test, while ^*^, ^**^ and ^***^ represent *p*‐value of < 0.05, < 0.01 and < 0.001, respectively.

In this study, the CRISPR/Cas9 knockout technique was introduced to investigate the role of the UPR pathway in regulating hybridoma antibody production. The functional hybridoma BFNB‐4E (wild type, WT) was selected for engineering due to its higher transfection efficiency, compared to BJQ‐E2 (data not shown). *Xbp1* (X‐box binding protein 1), an important gene downstream of the UPR pathway, was selected as the target (Figure 3A). An sgRNA was designed to target the first exon of *Xbp1s (*a spliced transcript of *Xbp1*) and integrated into the CRISPR/Cas9 vector for knockout (Figure 3B). A monoclonal mutant was screened (BFNB*^Xbp1s−^*), with significant down‐regulation of *Xbp1s* mRNA, compared to the WT (Figure 3C). Sanger sequencing found that the target sequence in BFNB*^Xbp1s−^* exhibited a frameshift mutation upstream of the PAM region of *Xbp1s* (Figure S6). According to Western blotting, no clear band was detected in WT, suggesting that XBP1s expression level may be below the quantification limit. Upon treatment with tunicamycin (TM, a well‐known ER stress inducer) for 8 h, a weak band was observed in WT, whereas it was abscent in TM‐induced BFNB*^Xbp1s^* ^−^ (Figure 3D). It not only confirmed the successful knockout of XBP1s in BFNB*^Xbp1s^* ^−^, but also demonstrated the effective activation of the UPR pathway using TM. As observed, *VH*, *VL, CH* and *CL* mRNA levels were significantly decreased in BFNB*^Xbp1s^* ^−^, in contrast to WT (Figure 3C). Additionally, both the expression level of secreted IgG protein (Figure 3E) and the productivity per single cell (q_mab_) (Figure 3F) were significantly down‐regulated. When staining with AF488‐labeled goat‐anti‐mouse IgG secondary antibody, the fluorescence intensity of BFNB*^Xbp1s^* ^−^ in the FITC channel was lower than that of WT, revealing a decreased amount of membrane‐localized IgG molecules (Figure 3G).

Furthermore, a rescue assay was conducted by resotring the expression of XBP1s in BFNB*^Xbp1s^* ^−^ (Figure 3H). As Western blot analysis depicted, the protein level of XBP1s was significantly increased using a vector overexpressing *Xbp1s*, indicating the effectiveness of rescue experiments and the specificity of the anti‐XBP1s antibody (Figure 3I). Moreover, the mRNA expression of *Xbp1s* was significantly up‐regulated in the engineered *Xbp1s* OE cells. Additionally, the mRNA levels of *VH* and *VL* were significantly increased (Figure 3J). In addition, the expression of secreted IgG protein in *Xbp1s* OE was also significantly enhanced (Figure 3K). The above results revealed that *Xbp1s* positively regulates Ig chain production in hybridomas, thereby uncovering the importance of the UPR pathway in modulating hybridomas antibody production.

### 2.4 CRISPR/HDR Editing of Hybridomas for Real‐Time Monitoring of UPR Activation

**FIGURE 4:**
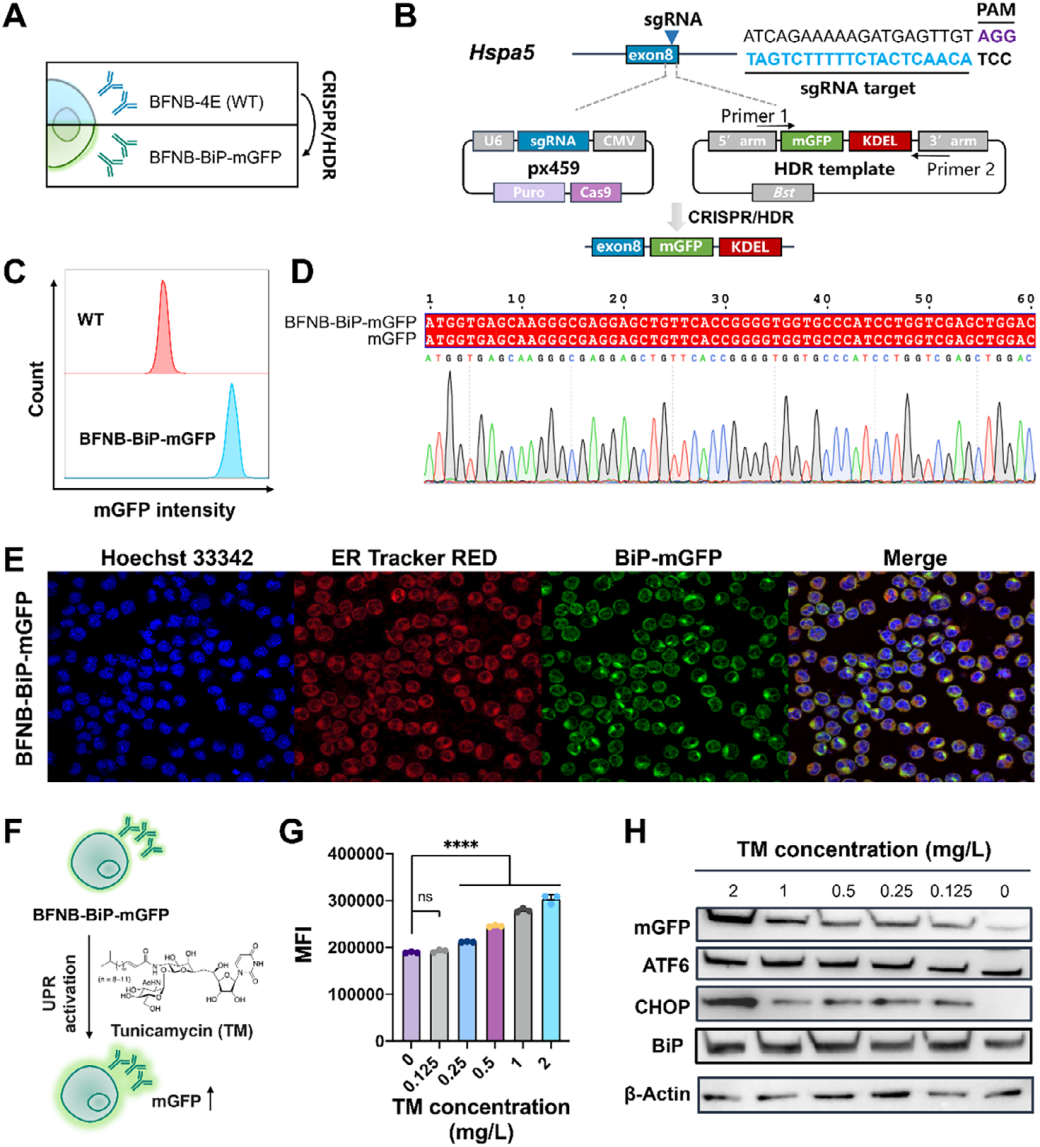
CRISPR/HDR engineering of hybridoma to construct an endogenous fluorescent system for monitoring UPR activation. (A) Schematic illustration of the CRISPR/HDR approach. A novel cell line, BFNB‐BiP‐mGFP, was developed using the hapten‐specific hybridoma BFNB‐4E (WT) for genomic editing. (B) The target exon 8 of *Hspa5*‐encoded BiP is shown. A Cas9 plasmid harboring an sgRNA targeting exon 8 was established, and the double‐stranded break was repaired by HDR using a donor vector consisting of mGFP, the KDEL motif and homology arms. (C) Flow cytometry analysis of mGFP signals in the engineered BFNB‐BiP‐mGFP cell line. The monoclonal of the target mutant was screened through antibiotic selection and limiting dilution. The WT was selected as the negative control. (D) Sanger sequencing of the amplified PCR product of mGFP. The DNA from BFNB‐BiP‐mGFP was utilized as the template for amplifying the mGFP sequence. (E) Confocal imaging of the BFNB‐BiP‐mGFP cell line stained with Hoechst 33342 nuclear dye and ER‐Tracker Red dye. (F) Activation of the UPR pathway in BFNB‐BiP‐mGFP using TM, a known ER stress inducer. (G) The median fluorescent intensities (MFIs) of BFNB‐BiP‐mGFP were recorded using flow cytometry after TM treatment at different concentrations (0.125, 0.25, 0.5, 1 and 2 mg/L, with 0.1% DMSO). Three biological replicates were performed, and error bars represent the standard deviation. Statistical significance was analyzed using Student's t‐test, while ns and **** represent *p*‐value of not significant and < 0.0001, respectively. (H) Western blotting analysis of UPR‐related proteins (ATF6, CHOP and BiP) and mGFP in BFNB‐BiP‐mGFP under TM exposure.

To further investigate the function of the UPR pathway in response to hybridoma homeostasis disruption, an intracellular reporter system was established using the CRIPSR/HDR technique. Here, a fluorescent mGFP marker was endogenously integrated into the *Hspa5* locus encoding BiP, an important ER chaperone (Figure 4A). An sgRNA was designed to target exon 8 region of *Hspa5* and then integrated into a Cas9 plasmid to create the double‐stranded break. According to the homology‐directed repair mechanism, the template harboring mGFP was integrated between the C‐terminal of the *Hspa5* locus and the upstream region of the KDEL ER retrieval motif, generating the functional fusion expression of BiP and mGFP (Figure 4B). The obtained monoclonal BFNB‐BiP‐mGFP cell line exhibited a higher fluorescence intensity at the FITC detection channel by flow cytometry, compared to the WT (BFNB‐4E) (Figure 4C). Additionally, the amplified PCR products from the BFNB‐BiP‐mGFP exhibited the correct sequence of mGFP, indicating successful gene insertion (Figure 4D). Laser confocal imaging further revealed the co‐localization of ER‐Tracker Red and the mGFP signal in the ER, demonstrating successful fusion expression of BiP and mGFP in BFNB‐BiP‐mGFP, a novel endogenous fluorescent reporter system (Figure 4E).

Furthermore, the capability of BFNB‐BiP‐mGFP to monitor UPR activation was examined by treating the cells with TM (Figure 4F). TM‐treated BFNB‐BiP‐mGFP cells were collected, and their mGFP intensities, as well as the expression levels of IgG proteins and antibody genes, were examined (Figure S7A). Based on tested TM concentrations (0.25–2 mg/L) that did not cause cytotoxicity (Figure S7B), we found that the addition of TM significantly enhanced intracellular mGFP intensities in BFNB‐BiP‐mGFP with a dose‐dependence manner (Figure S7C; Figure 4G). Compared to 0.1% DMSO, the mRNA levels of UPR‐related genes (including *BiP*, *Xbp1s*, *Ire1a*, *Atf6* and *Chop)* were significantly increased by 4.6–7.4‐fold, when treated with 0.25 mg/L TM (Figure S7D). This TM treatment also increased the protein levels of mGFP, ATF6, CHOP and BiP, indicating the activation of the UPR pathway in BFNB‐BiP‐mGFP (Figure 4H). The above results demonstrated that CRISPR/HDR‐based engineering of BFNB‐BiP‐mGFP was successful, in which UPR activation can be effectively monitored in real time by recording intracellular mGFP signals.

### 2.5 Correlation between UPR Activation and Disruption of IgG Production in Hybridomas

**FIGURE 5:**
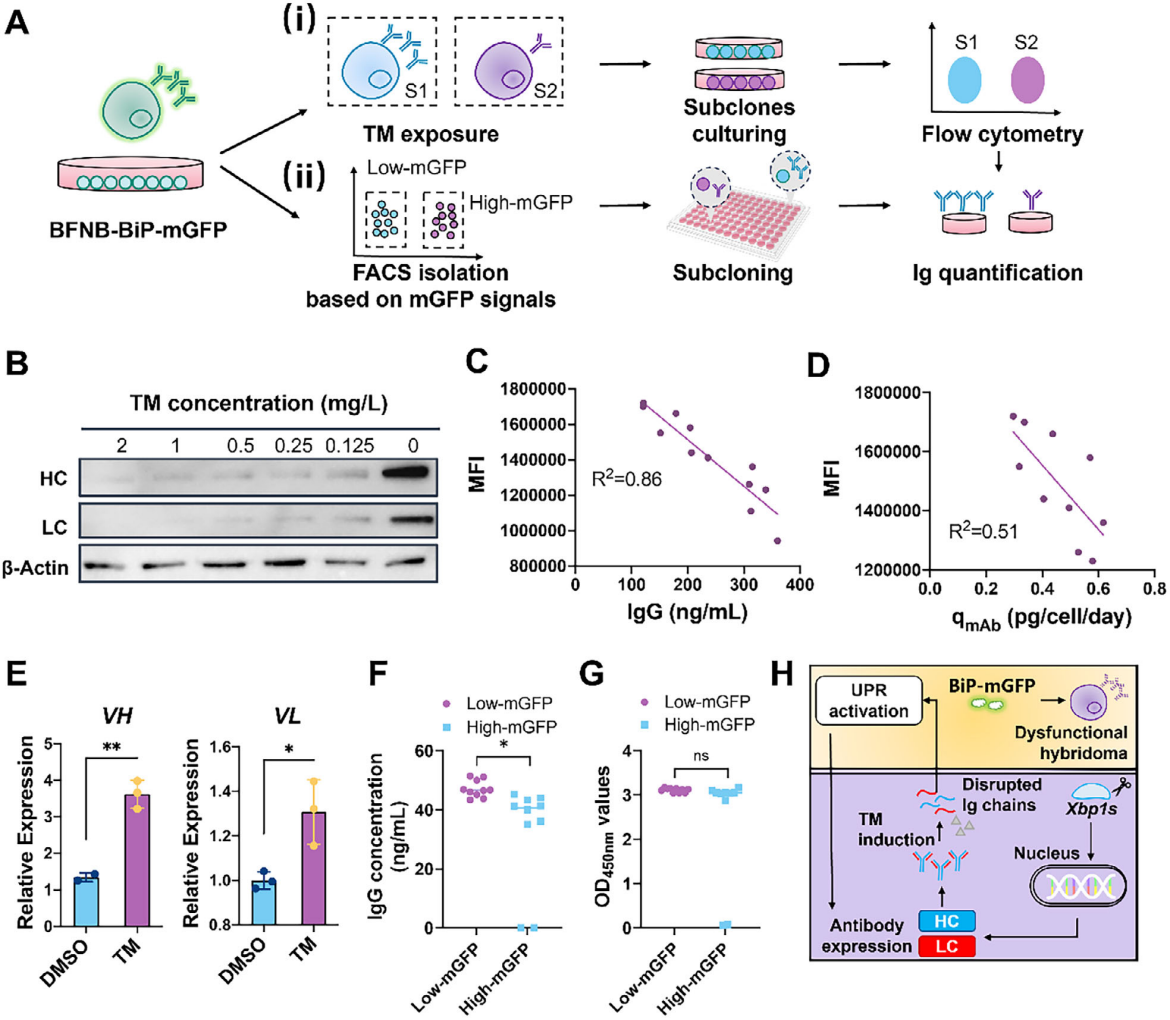
Relationship between UPR activation and Ig chain production in hybridomas. (A) Schematic illustration. (A‐i) Monitoring of disrupted Ig production upon TM exposure. The BFNB‐BiP‐mGFP cell line was treated with various concentrations of TM, and the resulting subclones were harvested for characterization. (A‐ii) Monitoring of Ig production in BFNB‐BiP‐mGFP subclones with distinct mGFP intensities. Subclones from the populations with high‐mGFP (> 8 × 10^4^) and low‐mGFP (< 2 × 10^4^) intensities were isolated into 96‐well plates via FACS. The intracellular mGFP intensities and IgG levels of each subclone were analyzed using flow cytometry and an IgG quantification kit. (B) Impact of TM on HC and LC expression. Total proteins from TM‐induced BFNB‐BiP‐mGFP cells were extracted and analyzed via Western blotting. Cells treated with 0.01% DMSO served as the control. (C,D) Correlation analysis between mGFP intensity and secreted IgG level (C), and between mGFP intensity and q_mab_ (D) upon TM induction. TM‐induced BFNB‐BiP‐mGFP subclones were used for assessment. (E) Expression levels of VH and VL mRNA in the BFNB‐BiP‐mGFP cell line upon TM induction (0.25 mg/L). Cells treated with 0.01% DMSO served as the control. Three biological replicates were performed, and error bars represent the standard deviation. (F,G) Measured IgG levels (F) and OD_450nm_ values (G) for BFNB‐BiP‐mGFP subclones isolated from low‐mGFP (n = 10) and high‐mGFP populations (n = 10). Subclones were seeded in 24‐well plates, and their supernatants were collected for analysis using the IgG quantification kit and antigen‐coated ELISA plates. Statistical significance was analyzed using Student's *t*‐test, while ns, ^*^ and ^**^ represent *p*‐value of not significant, < 0.05 and < 0.01, respectively. (H) Roles of the UPR pathway in facilitating antibody production and monitoring disrupted IgG production in hybridomas. The UPR pathway activator, *Xbp1s*, positively enhances hybridoma antibody expression at both the mRNA and protein levels. TM‐induced loss of HC and LC leads to impaired IgG production in hybridomas, which in turn activates the UPR pathway and increases mGFP intensity. Conversely, a stronger mGFP signal, indicating UPR activation, suggests the presence of dysfunctional hybridomas.

TM is a well‐known UPR inducer that inhibits N‐linked glycosylation of ER client proteins. In principle, the application of TM interferes with Ig chain production and disrupts homeostasis of BFNB‐BiP‐mGFP, which can then be monitored by analyzing IgG protein levels and recording intracellular mGFP intensities (Figure 5A). Compared to the WT (BFNB‐4E), BFNB‐BiP‐mGFP exhibited a similar level of IgG expression (150 ng/mL) and a calculated q_mab_ of 0.25 pg/cell/day, indicating the insertion of the exogenous mGFP tag did not affect antibody production in hybridomas. Under TM exposure (0.25–2 mg/L), the detected IgG levels in BFNB‐BiP‐mGFP (54.0–109.65 ng/mL) were dramatically decreased with statistical significance, in contrast to the 0.1% DMSO control (148.02 ng/mL) (Figure S7E). A similar trend was observed in TM‐induced WT cells (Figure S7F). Meanwhile, expression of both HC and LC in BFNB‐BiP‐mGFP decreased after TM exposure (0.125–2 mg/L) (Figure 5B). This revealed the negative impact of TM on hybridoma Ig production, which is consistent with its known mechanism of action. By monitoring TM‐induced subclones, we observed that intracellular mGFP intensities gradually increased as Ig production was disrupted due to TM‐triggered artificial UPR stress. Additionally, a strong negative correlation was found between IgG levels and MFIs (R^2^ = 0.86) (Figure 5C), as well as between q_mab_ and MFIs (R^2^ = 0.51) (Figure 5D). We inferred that the weaker correlation between q_mab_ and MFI might be caused by changes in the number of viable cells induced by UPR activation, or by the selection of the variable range used for curve fitting. Despite these differences, the overall trend remains clear and reliable, demonstrating the negative correlation between IgG production and UPR activation. These results demonstrate the accuracy and effectiveness of the engineered BFNB‐BiP‐mGFP in monitoring disrupted Ig production under TM induction. Despite the reduction at the protein levels, TM induction (0.25 mg/L) significantly increased the mRNA expression levels of VH (3.6‐fold) and VL (1.3‐fold) in BFNB‐BiP‐mGFP, compared to the negative control (0.1% DMSO) (Figure 5E). Additionally, the application of TM also upregulated VH and VL mRNA levels in WT cells (Figure S7G). The underlying mechanism requires further investigation.

Additionally, we investigated the feasibility of this novel hybridoma‐based intracellular fluorescence reporter system for tracking antibody production stability. Based on appropriate gating (Figure S8A–C), high‐mGFP (Fluorescence intensity > 8×10^4^) and low‐mGFP (< 2 × 10^4^) subpopulations were identified from BFNB‐BiP‐mGFP through FACS, with frequencies of 3.8% and 2.7%, respectively (Figure S8D,E). In total, 20 subclones were isolated from the high‐mGFP (n = 10) and low‐mGFP (n = 10) subpopulations, and their IgG secretion levels were detected (Figure 5A). Compared to 10 subclones collected from the low‐mGFP group (Figure S8F), 10 subclones from the high‐mGFP subpopulation exhibited lower IgG concentrations (Figure S8G). Two dysfunctional subclones (H3‐1D and H4‐6D) were newly screened from the latter group, which showed extremely low absorbance values even below the IgG quantification limit. In contrast, low‐mGFP subclones exhibited an average IgG concentration of 47.1 ng/mL, significantly (*p* < 0.05) higher than that of the high‐mGFP subclones (with an average IgG concentration of 32.6 ng/mL) (Figure 5F). In addition, we examined the recognition specificity of these subclones using non‐competitive ELISA. No statistically significant difference was observed between the low‐mGFP and high‐mGFP subpopulations in terms of OD_450nm_ values (Figure 5G). All low‐mGFP subclones retained specific recognition toward the coated antigen, with OD_450nm_ values ranging from 3.04 to 3.17 (Figure S8F). For the isolated high‐mGFP subclones, the two newly identified dysfunctional hybridomas (H3‐1D: 0.06 and H4‐6D: 0.07) exhibited markedly lower OD_450nm_ values, compared to the other eight subclones with signals ranging from 2.87 to 3.17 (Figure S8G). These results demonstrate that the engineered BFNB‐BiP‐mGFP cell line is feasible for isolating functional and productive hybridomas through FACS, with higher mGFP intensities potentially indicating the presence of dysfunctional hybridomas. Since UPR levels (as indicated by mGFP intensity) can be monitored depending on the definition and type of disrupted Ig production, BFNB‐BiP‐mGFP cell line also provides a valuable tool for enhancing our understanding of antibody homeostasis in hybridomas.

In summary, we identified Ig chain loss in dysfunctional hybridomas through traditional limiting dilution and BCR‐Seq. Proteomic sequencing revealed the important role of the UPR pathway in regulating hybridoma homeostasis and facilitating antibody production. This was further validated through CRISPR/Cas9 knockout and rescue experiments by targeting *Xbp1s*, an activator of the UPR pathway. Based on CRISPR/HDR editing, a novel fluorescent reporter system was established to monitor UPR activation. According to mGFP signal monitoring and IgG quantification, we revealed that TM induction disrupted Ig chain production in the engineered BFNB‐BiP‐mGFP cell line, leading to UPR activation and enhanced mGFP signal. A strong negative correlation was observed between mGFP intensities and IgG levels. Additionally, dysfunctional hybridomas were effectively isolated by subcloning cells with higher mGFP signals. These results demonstrate the effectiveness of the BFNB‐BiP‐mGFP cell line in tracking disrupted Ig production and hybridoma homeostasis by monitoring UPR activation (Figure 5H).

## 3 Discussion

Homeostasis disorder in antibody‐secretory cells poses a significant threat to achieving sustainable antibody production. Earlier studies have highlighted the substantial impact of Ig chain loss on hybridoma homeostasis and antibody quality control (3, 4, 30). Through limiting dilution cloning, our characterization indicated that the loss of HC production, as well as the simultaneous loss of both HC and LC, is associated with the emergence of dysfunctional hybridomas that lack hapten‐specific recognition and IgG secretion. However, we did not identify any BJQ‐derived subclones that lack only the LC (HC^+^/LC^−^), even among two other hybridoma cell lines, including PA‐1A6 (which secretes IgG against parathion, a representative organophosphorus insecticide) and BFNB‐4E (which secretes IgG against carbofuran, a representative carbamate insecticide) (data not shown here). These findings indicated a preferential spontaneous loss of HC production rather than LC in hybridomas, supporting the fundamental hypothesis of HC toxicity as previously reported [3, 30, 31]. Furthermore, they highlighted the challenges associated with screening subclones that produce HC without the LC partner. Hence, we envisioned that targeted deletion or knockdown of the LC gene may help clarify the relationship between LC loss alone and hybridoma homeostasis disruption, as well as the link between excessive HC production and UPR regulation. By profiling Ig transcripts, we found that active VH mRNA encoding hapten‐specific antibodies remained in two HC‐deficient hybridomas (BJQ‐D4/1C10), but was undetectable in another subclone that failed to produce HC (BJQ‐E7). Interestingly, the target VH and VL sequences were simultaneously ranked as the most highly expressed mRNAs in both the functional BJQ‐E2 and HC‐deficient BJQ‐D4. These results demonstrated the feasibility of high‐throughput BCR‐Seq for identifying effective Ig genes even in hybridomas that fail to secrete hapten‐specific antibodies, as reported in our previous study [32], although another study did not support this observation [17]. In addition, our findings suggested that the underlying causes of aberrant Ig production, particularly the loss of HC, are likely highly complex and diverse. Previously, the loss of antibody productivity in mammalian cells (e.g., CHO) has been associated with histone hypoacetylation [33], DNA methylation [34] and telomere stasis [35, 36]. A recent study revealed a key role for post‐transcriptional RNA modification of *Ighg1* in maintaining IgG1 homeostasis in antibody‐secreting B cells [28]. Therefore, we inferred that post‐transcriptional downregulation or transcriptional suppression of VH mRNA may contribute to HC loss in hybridomas, which still requires further investigation.

As a hallmark of protein homeostasis disorder, the loss of HC in secretory cells (e.g., hybridomas and plasma cells) has led to the hypothesis that the intracellular accumulation of misfolded HCs induces cellular toxicity by triggering ER stress and activating the UPR pathway, which are essential components of the cellular protein quality control system [3, 29, 30]. In Ig‐secreting plasma cells, the accumulation of unpaired intracellular HCs resulting from LC knockdown activates the UPR and ERAD pathways, revealing the relationship between Ig chain imbalance and ER stress induction [37]. Using naturally screened dysfunctional BJQ‐D4 (HC^−^/LC^+^) and functional BJQ‐E2 (HC^+^/LC^+^) subclones for high‐throughput sequencing, our proteomics analysis revealed that ER stress and the UPR pathway play crucial roles in maintaining hybridoma stability. These findings addressed the knowledge gap between HC loss and ER stress in hybridomas and motivated us to explore whether targeting the UPR pathway could help regulate antibody production. *Xbp1*, a transcriptional factor located downstream of the IRE1α/XBP1 branch of the UPR pathway, is necessary for plasma cell differentiation [38] and high‐level Ig production within the secretory apparatus [24]. The differentiation potential of *Xbp1*‐deficient B cells can be successfully restored by *Xbp1s*, the spliced and active form produced through UPR activation [39]. In this study, we found that *Xbp1s* knockout led to decreased IgG expression in hybridomas, while restoring *Xbp1s* expression reversed this effect, indicating the essential role of the UPR pathway in regulating hybridoma antibody production. As one of the most important UPR sensors, BiP is an ER chaperone that plays a central role in UPR activation by dissociating from transmembrane proteins such as IRE1α, PERK and ATF6 [40, 41]. Previous studies have reported the functions of BiP in influencing UPR activation, maintaining ER homeostasis [42], and affecting IgG expression in CHO cells [25, 26]. Earlier reports also demonstrated a positive correlation between HC expression and UPR activation in antibody‐producing CHO cells [25, 26] and human HeLa cells [42]. In this context, the lower UPR levels observed in BJQ‐D4 compared with BJQ‐E2 are likely attributed to its lack of HC. Whether naturally disrupted Ig chain production (e.g., the loss of HC or LC) influences hybridoma homeostasis through UPR activation still requires further confirmation. Targeted deletion of Ig chains could help clarify whether and how Ig chain loss specifically triggers UPR activation and its adaptive mechanisms. For functional hybridomas expressing both HC and LC, we first identified a negative correlation between TM‐induced UPR activation and IgG production using an intracellular fluorescence reporter system, BFNB‐BiP‐mGFP. Interestingly, TM‐induced UPR activation increased antibody mRNA expression while decreasing IgG protein levels, suggesting a possible compensatory mechanism whereby the UPR signaling threshold regulates antibody quality control. Previous studies have shown that the ER‐associated protein degradation (ERAD) pathway alleviates ER stress and maintains UPR homeostasis by clearing misfolded proteins or antibodies [43, 44, 45]. We therefore hypothesize that TM‐induced aberrant Ig chain production activates the UPR, which in turn stimulates the ERAD pathway to degrade misfolded Ig chains. It is also possible that the increase in Ig transcripts may result from an adaptive UPR mechanism aimed at restoring functional Ig production under ER stress. As different stages of UPR‐induced ER stress exhibit opposing functions [46], our results suggest that the magnitude of ER stress signaling could potentially reflect distinctive stages of hybridoma homeostasis.

Maintenance of hybridoma homeostasis is essential for in vitro antibody cultivation and engineering. By targeting the UPR pathway, this fluorescence reporter BFNB‐BiP‐mGFP cell line reflects Ig production by monitoring intracellular mGFP signals. Notably, hybridoma subclones exhibiting stronger mGFP signals were found to be dysfunctional, validating its effectiveness and reliability for isolating hapten‐specific antibodies. Compared with conventional FACS‐based enrichment, the new system eliminates the need for fluorescent probe fabrication and tedious staining procedures, enabling dynamic monitoring of antibody production and non‐invasive analysis of UPR activation in secretory cells. Previously, novel UPR‐based probes [47, 48] and ER proteome profiling system [49, 50] have been successfully established to assess UPR status at a point‐of‐end. As an alternative, this cell line enables real‐time, in situ and continuous detection of UPR activation during long‐term cultivation. Despite the fact that engineering hybridoma cell lines using CRISPR/Cas9 remains time‐consuming and labor‐intensive, particularly with respect to plasmid construction and monoclonal characterization, we suggest that the introduction of FACS for isolating target fluorescent mutants can improve the efficiency of the overall process. Moreover, a previous study has demonstrated that the extent of UPR‐induced activation was associated with cell performance (e.g., viability and growth) and cell culture parameters (e.g., medium composition and osmolarity) in antibody‐producing CHO cells [26]. Thus, we envisioned that the newly established BFNB‐BiP‐mGFP cell line could accelerate the optimization of the cell culture environment, thereby facilitating the selection of high‐producing clones in both fed‐batch and bioreactor systems in future studies. Elucidating the mechanisms linking UPR activation to antibody production will provide in‐depth insights into the regulation of hybridoma homeostasis and may inform strategies to improve antibody stability. As different hybridomas may exhibit distinct genomic profiles, whether the correlation between UPR activation and Ig chain production can be observed in other antigen‐specific hybridoma cell lines still requires further investigation.

## 4 Experimental Section

### 4.1 Hybridoma Cell Culture

Through hybridoma technology, hapten‐specific BJQ‐FS1 (a hybridoma secreting monoclonal antibodies against chlorothalonil, a representative fungicide) and BFNB‐4E (a hybridoma secreting monoclonal antibodies against carbofuran, a representative insecticide) [7], which were previously developed in our laboratory, were selected as model cell lines in this study. These mycoplasma‐free cell lines have not been assigned with RRIDs and are not commercially available. The hybridomas were cultured in Dulbecco's Modified Eagle Medium (DMEM) (Meilune, Dalian, China), supplemented with 10% heat‐inactivated fetal bovine serum (FBS) (Catalog#13011‐8611, Tianhang, Hangzhou, China) and 1% Penicillin‐Streptomycin solution (Catalog#15140122, Gibco, Carlsbad, USA). The cells were kept in the incubator (37°C, 5% CO_2_).

### 4.2 Subclone Screening

Functional and dysfunctional subclones of the BJQ‐FS1 cell line that were retrieved from −80°C after approximately one year of preservation, were collected through limiting dilution screening. The binding specificity of the identified monoclonal subclones was then characterized via a non‐competitive ELISA (nc‐ELISA), according to previous studies with slight modifications [51]. First, the coating antigen (BJQ‐OVA, 10 mg/L, 100 µL/well) was immobilized in 96‐well plates. The structures of pesticide and its hapten were illustrated in the Supporting Information. After an overnight incubation at 4°C, the plates were blocked with 2% milk‐PBS (0.01 m PBS, pH 7.4, containing 2% milk powder) for 30 min at 37°C. Next, the supernatants of each subclones (100 µL/well) were added into the plate and incubated at 37°C for 1 h. Then, the plate was washed three times using PBST (0.01 M PBS, pH 7.4, containing 0.2% Tween‐20 (Catalog#P2287, Sigma Aldrich, Saint Louis, USA)). Next, the plates were incubated with HRP‐labeled goat‐anti‐mouse IgG (H+L) secondary antibody (Catalog#AP308P, Sigma Aldrich, Saint Louis, USA) at 37°C for 1 h. Then, the plates were washed with PBST for five times and added with 3,3’,5,5’‐Tetramethylbenzidine (TMB) solution (100 µL/well). After an incubation at 37°C for 15 min, each well was added with 2 m H_2_SO_4_ (50 µL/well) and the absorbance at 450 nm (OD_450nm_) was measured with SpectraMax i3 (Molecular Devices, San Jose, USA). Finally, functional and dysfunctional hybridoma subclones were identified based on OD_450nm_ >1 and OD_450nm_ <0.1, respectively.

### 4.3 Hybridoma Characterizations

Hybridomas (1 × 10^6^) protein was obtained according to the protocol by using the ProteinExt Mammalian Total Protein Extraction Kit (Catalog#DE101‐01, TransGen, Beijing, China). The concentration of extracted protein was determined with BCA method by using an Easy II Protein Quantitative Kit (Catalog#DQ111‐01, TransGen, Beijing, China). The expression levels of IgG molecules from the secreted supernatants and the extracted protein (50 µg) were analyzed using a Mouse IgG ELISA Kit (Catalog#E‐EL‐M0692, Elabscience, Wuhan, China), according to the manufacturer's protocol. Hybridoma supernatants were collected and their isotypes were determined using an HRP‐labeled goat‐anti‐mouse heavy chain (IgG1/G2a/G2b/G3/M/A) and light chain (κ/λ) ELISA kit (Catalog#BF06001, Biodragon, Suzhou, China). The expression of HC and LC chains of different subclones were examined through Western blotting by staining with HRP‐labeled goat‐anti‐mouse IgG (H+L) secondary antibody (Catalog#AP308P, Sigma Aldrich, Saint Louis, USA).

### 4.4 B‐Cell Receptor Repertoire Sequencing (BCR‐Seq)

The distribution of VR mRNA in varied BJQ‐derived subclones was traced using high‐throughput BCR‐Seq. First, total RNA was isolated using RNAiso Plus reagent (Catalog#9108, Takara, Beijing, China). Then, VR libraries were constructed via the ImmuHub BCR amplification system (ImmuQuad Biotech, Hangzhou, China). Accordingly, the VR mRNAs were amplified using a universal forward primer and isotype‐specific reverse primers for heavy chain (IgG, IgA, IgM, IgD and IgE) and light chain (Igκ and Igλ) via the 5’‐RACE technique. The constructed VH and VL libraries were sequenced using the Illumina HiSeq system (San Diego, USA) with 150 bp paired‐end sequencing. According to bioinformatic analysis, the V/D/J classification, their encoding amino sequence and the complementarity‐determining region 3 (CDR3) of clean reads were obtained using the NCBI (http://www.ncbi.nlm.nih.gov/) database. Meanwhile, the effective VR sequences encoding hapten‐specific antibodies against chlorothalonil were obtained from a functional subclone through V‐region Sanger sequencing, and then validated using the HEK293(F) mammalian cell expression system, according to our previous studies [7, 51, 52]. The clone counts and their fraction rates of all the sequenced VRs were calculated.

### 4.5 Proteome Profiling with LC‐MS/MS

The total protein of subclones was extracted and quantified, then pretreated with reductive alkylation using triethylammonium bicarbonate (TEAB) and tris(2‐carboxyethyl) phosphine (TCEP). After digestion, the dehydrated peptides were redissolved in 0.1% trifluoroacetic acid (TFA) solution and desalinated. The peptides were then analyzed in an LC‐MS/MS system consisting of an EASY‐nLC 1200 system and a timsTOF Pro2 MS/MS (Bruker, Bremen, Germany), equipped with an C18 column (75 µm × 25 cm, IonOpticks, USA). A gradient was applied with mobile phases A: 2% acetonitrile and 0.1% formic acid, and B: 80% acetonitrile and 0.1% formic acid (90/10, v/v). MS/MS detection was conducted using the following settings: electrospray voltage 1.5 kV, 100–1700 m/z, collecting mode is the Parallel Accumulation‐Serial Fragmentation (PASEF), with full scanning and ten PASEF MS/MS scans. MaxQuant software (version 2.0.3.1) was employed for peptide analysis (database: Mus_musculus.GRCm38.pep.all.fa_unique.fasta). The detailed parameters are listed in Table S4. In this study, Gene Set Enrichment Analysis (GSEA) was performed to determine essential pathways and terms by calculating the enrichment score. Based on the quantified abundance, differentially expressed proteins (DEPs) were identified with the criteria of |Log2Foldchange| ≧1, with a *p*‐value <0.05. Gene Ontology (GO) enrichment analysis was then conducted to identify key pathways involved in antibody production and hybridoma homeostasis using the databased (http://geneotology.org/). Protein‐protein interaction (PPI) analysis was performed using the String website (https://string‐db.org/).

### 4.6 Construction of CRISPR/Cas9 Knockout Plasmid

The *XBP1* was selected as the target for genomic knockout using CRISPR/Cas9 technique, according to previous reports with slight modifications [9, 53]. First, the sgRNA (5’‐GACACCGCAGGCTCGCAAG‐3’) of *Xbp1s* was designed using an online tool from Zhang laboratory (http://crispr.mit.edu), which was synthesized with overhangs for cloning (Sangon, Shanghai, China). Next, the oligos were phosphorylated with T4 PNK (New England Biolabs, Ipswich, MA, USA) and then annealed at 95°C for 5 min in a thermocycler. The annealed oligos were gradually cooled to 25°C (4°C/min) and then cloned into the backbone of pSpCas9(BB)‐2A‐Puro (PX459) V2.0 (Addgene #62988, Watertown, MA, USA) by incubating at room temperature (RT) for 20 min. Using Dh5α *E. coli*, the endotoxin‐free sgRNA/Cas9 plasmid was prepared and further purified using NucleoBond Xtra Midi Kit (Catalog#740410, Macherey‐Nagel, Düren, Germany). The target sgRNA of interest was validated through Sanger sequencing (pxSeq primer: 5’‐CGTAACTTGAAAGTATTTCGATTTCTTGGC‐3’).

### 4.7 Construction of CRISPR/HDR Knock‐in Vectors

To establish a fluorescence system for monitoring UPR activation, the fluorescent protein mGFP was inserted into exon 8 of endogenous *BiP* (also known as *Hspa5*) using CRISPR/HDR. First, the sgRNA (5’‐TAGTCTTTTTCTACTCAACA‐3’) of *BiP* was designed, and then the sgRNA/Cas9 plasmid was constructed as described above. According to a previous study with slight modification [25], the homology‐directed repair (HDR) template containing the sequences of mGFP and KDEL was designed and synthesized (Sangon, Shanghai, China). The homology arms were synthesized according to previous literature with slight modifications [54]. Next, the HDR plasmid was constructed by integrating the HDR template into the vector pUC‐GW and the endotoxin‐free HDR plasmid was then prepared as described above.

### 4.8 Hybridoma Nucleofection

The established CRISPR/Cas9 and CRISPR/HDR vectors were transfected into hybridomas via nucleofection, according to previous studies with slight modifications [9, 53]. In this study, hapten‐specific hybridoma BFNB‐4E was selected for engineering. First, hybridomas (3 × 10^4^ cells) were collected via centrifugation (500 g, 5 min, 4°C) and then resuspended in 100 µL SF cell line medium (Catalog#V4XC‐2012, Lonza, Basel, Switzerland). Afterward, 1 µg CRISPR/Cas9 plasmids and 1 µg HDR vectors, or 2 µg of CRISPPR/Cas9 were added into the cell suspension. After gentle mixing, the mixture was transferred into cuvettes (Catalog#V4XC‐2012, Lonza, Gampel, Switzerland) and then nucleofection was performed according to the CQ‐104 program, Cell line CF by using 4D‐Nucleofection System (Lonza, Basel, Switzerland). The transfected cells were subsequently transferred to a six‐well plate with 3 mL cell culturing medium and cultured in the incubator (37°C, 5% CO_2_).

### 4.9 Screening of Engineered Mutants

For CRISPR/Cas9 knockout, antibiotic screening was conducted by culturing the transfected hybridomas with screening medium (general cell culture medium containing 5.5 mg/L puromycin, Thermo Fisher, Waltham, USA) for 3–5 days. The medium was refreshed daily. The surviving cells were then collected, and the monoclonal engineered hybridoma (BFNB*^Xbp1s−^*) was obtained through limiting dilution screening. For CRISPR/HDR knock‐in, the transfected hybridomas were cultured and screened as described above. The viable cells were collected, expanded and then treated with another screening medium (general cell culture medium containing 7 mg/L blasticidin, Yeasen, Shanghai, China) for an additional 7 days. According to the limiting dilution method, the monoclonal knock‐in mutant (BFNB‐BiP‐mGFP) was obtained, and its fluorescence intensity was analyzed by flow cytometry. Finally, two collected mutants were expanded in 75 cm^2^ flasks and stored in nitrogen for long‐term preservation.

### 4.10 DNA Extraction and Sequencing

The knockout and knock‐in efficiency were determined by amplifying the target sequences of interest via PCR. First, the DNA of the engineered mutants was extracted using the MiniBEST Universal Genomic DNA Extraction Kit Ver.5.0 (Catalog#9765, Takara, Beijing, China), according to manufacturer's instructions. Afterward, PCR amplification was performed using the 2×*TransStart FastPfu* PCR SuperMix (Catalog#AS231‐01, Transgen, Beijing, China). The PCR was conducted as follows: 2 min at 95°C, 30 cycles of 20 s at 95°C and 1 min at 72°C, 5 min at 72°C. The sequences of interest for Xbp1s and BiP‐mGFP were amplified using the primers listed in Table S5. Finally, the amplified DNA products were cloned into the pEASY‐Blunt Zero Cloning Vector (Catalog#CB501‐01, Transgen, Beijing, China) and confirmed via Sanger sequencing (primer: 5’‐TATTGTCCAGATTGTGTGCAGCCATATG‐3’).

### 4.11 qRT‐PCR

First, total RNA was extracted with RNAiso Plus and then transcribed into cDNA using PrimeScript RT Master Mix (Catalog#RR036A, Takara, Beijing, China). Using the synthesized cDNA as the template, qPCR was performed on the QuantStudio 3 Real‐Time PCR system (Thermo Fisher, Waltham, USA), according to the SYBR Green PCR Master Mix (Catalog#2307541, Takara, Beijing, China). The running procedure was as follows: 2 min at 50°C, followed by 10 min at 95°C, and then 40 cycles of 15 s at 95°C and 1 min at 60°C. The melting curve program was as follows: 95°C for 15 s, 60°C for 1 min, and 95°C for 1s. GADPH was used as the internal standard. All primers are listed in Table S6. The data were analyzed using the ΔΔCt method.

### 4.12 Western Blotting

The extracted total protein was separated by SDS gel electrophoresis (180 V, 30 min, Bio‐Rad Laboratories, California, USA) and transferred to the 0.22 µm polyvinylidene difluoride (PVDF) membrane using the eBlot L1 Fast Wet Transfer System (Catalog#L00686, GenScript, Nanjing, China). Next, the membrane was blocked with PBS (0.01 m, pH 7.4, containing 5% skim milk powder) at RT for 1 h, and then washed with PBST (0.01 m, pH 7.4, containing 0.05% Tween‐20) three times (with an interval of 10 min). The membrane was then incubated with the HRP‐labeled goat‐anti‐mouse IgG secondary antibody (1:10000) (Catalog#AP308P, Sigma Aldrich, Saint Louis, USA), or the anti‐XBP1 antibody (Cataolog #ab37152, Abcam, Cambridge, USA), anti‐mGFP antibody (Catalog#ab290, Abcam), anti‐ATF6 (Catalog#ab37149, Abcam), anti‐DDIT3 (Catalog#ab11419, Abcam), anti‐GRP78 BiP antibody (Catalog#ab21685, Abcam). Prior to detecting XBP1s protein level, the hybridomas were treated with tunicamycin (0.25 mg/L, 0.1% DMSO) for 8 h. The anti‐β‐Actin antibody (Catalog#GB12001‐100, Servicebio, Wuhan, China) was used as the internal control. After overnight incubation at 4°C and washing three times with PBST, the membrane was incubated with SuperSignal West Atto Ultimate Sensitivity Substrate (Catalog#A38554, Thermo Fisher, Waltham, USA) and then detected by chemiluminescent gel imaging system (Bio‐Rad Laboratories, California, USA).

### 4.13 Flow Cytometry

The levels of membrane‐located IgG molecules of the engineered BFNB*^Xbp1s−^* and BFNB‐4E (wildtype, WT) were compared using flow cytometry. Briefly, hybridomas were collected with centrifugation (500 g, 5 min, 4°C) and washed with sterile PBS (0.01 M, pH 7.4) twice. Afterward, the cell pellets were stained with the 100 µL AF488‐labeled goat‐anti‐mouse IgG (H+L) secondary antibody (Catalog#A11001, Invitrogen, Carlsbad, USA, 2 mg/L), in staining buffer (sterile PBS containing 2% FBS). Subsequently, cell suspensions were loaded to CytoFLEX (Beckman Coulter, Indiana, USA) after filtration with 0.22 µm membrane, and their fluorescence intensities from the FITC channel were measured using CytExpert software (Beckman Coulter, Indiana, USA). A total of 10,000 events were recorded at a flow rate of 10 µL/min. The visualization was conducted using FlowJo software 8.0 (Ashland, Oregon, USA), and the median fluorescence intensities (MFIs) of identified cell populations were calculated after gating.

In addition, functional and dysfunctional subclones of BJQ‐derived and BFNB‐derived hybridomas were selected to investigate the role of the ER in antibody production. After staining with ER‐Tracker Red (Catalog#C1041S, Beyotime, Beijing, China), the fluorescence spectrum of each subclone in the PE channel was recorded by CytoFLEX, and their MFIs were calculated using FlowJo, respectively.

### 4.14 Rescue Experiment

The function of *Xbp1s* in regulating hybridoma antibody production was examined in this study. First, overexpression vector pcDNA3.1, which contains the cDNA sequence of *Xbp1s*, was synthesized by GenePharma (Shanghai, China). Then, mutant BFNB*^Xbp1s−^* (3 × 10^4^ cells) were transfected with the overexpression vector using jetPRIME reagent (Catalog#101000001, PolyPlus, Illkirch, France), according to manufacturer's instructions. Briefly, 1 µg of DNA was diluted in 200 µL of jetPRIME buffer. After vigorous vertexing for 10 s, 2 µL of jetPRIME buffer was added to the mixture and then incubated for 15 min at RT. Subsequently, the mixture was added dropwise to the cells. In 24 h post‐incubation, the efficiency of overexpression was monitored by examining expression level of *Xbp1s* mRNA and XBP1s protein in *Xbp1s* OE and BFNB*^Xbp1s−^* cell lines via qRT‐PCR and Western blotting, respectively.

### 4.15 Confocal Laser Scanning

The engineered mutant of BFNB‐BiP‐mGFP was seeded into a 6‐well plate and then stained with ER‐Tracker Red and Hoechst 33342 (Catalog#C1022, Beyotime, Beijing, China), according to manufactures’ instructions. After incubating at the 37°C incubator for 30 min, the hybridomas were washed three times with sterile PBS and then visualized with confocal laser scanning microscope FV3000 (Olympus, Tokyo, Japan).

### 4.16 Monitoring of UPR Activation

The effectiveness of the engineered BFNB‐BiP‐mGFP for monitoring UPR activation was assessed by treating with different levels of tunicamycin (TM), a well‐known ER stress inducer. First, hybridomas (1 × 10^6^ cells) were exposed to various concentrations of TM (0, 0.125, 0.25, 0.5, 1, 2 mg/L, 0.1% DMSO) in general cell culture medium for 24 h. The cytotoxicity of TM in hybridomas was examined using a Cell Counting Kit‐8 kit (Catalog#C0048S, Beyotime, Beijing, China) according to manufacturer's instructions. The levels of secreted IgG in BFNB‐BiP‐mGPF‐TM and WT (BFNB‐4E) were quantified as described above. After exposure, the mGFP signals of different TM‐induced subclones (BFNB‐BiP‐mGPF‐TM) in FITC channel were recorded using CytoFLEX. Furthermore, the expression levels of UPR‐related signaling cascades and Ig molecules of BFNB‐BiP‐mGPF‐TM were analyzed through qRT‐PCR and Western blotting, respectively.

### 4.17 Effects of UPR Activation on Ig Production

The Ig production levels of the engineered hybridomas (BFNB*^Xbp1s−^*, XBP1s OE and BFNB‐BiP‐mGFP) and the TM‐induced subclones (BFNB‐BiP‐mGPF‐TM) were compared in this study. Following RNA extraction and cDNA synthesis, the transcriptional levels of antibody mRNA (*VH*, *VL*, *CH* and *CL*) were examined using qRT‐PCR. Meanwhile, the expression levels of HC and LC proteins were determined via Western blotting. The concentrations of secreted IgG molecules were measured as described above. According to a previous study, the antibody productivity was determined as q_mab_ as follows: q_mab_ (pg/cell/day) = amount of IgG / [(*N*‐*N_0_*) × *t*/ ln(*N*/*N_0_*)], where *N* and *N_0_* represent the number of viable cells at the beginning and end of the testing period, respectively, and *t* stands for the number of days in the interval [55]. Moreover, the intracellular fluorescence intensities of BFNB‐BiP‐mGFP‐TM subclones were recorded through FITC channel using CytoFLEX. Finally, the linear correlation between mGFP signals and secreted IgG levels, as well as q_mab,_ was fitted using Graphpad Prism software 8.0 (Dotmatics, Boston, USA).

### 4.18 Correlation Between UPR Activation and Antibody Production

The subclones of the engineered BFNB‐BiP‐mGFP cell line with high mGFP intensity (High‐mGFP, > 8×10^4^) and low mGFP intensity (Low‐mGFP, < 2 × 10^4^) were isolated into 96‐well plates using the BD FACSAria Fusion Flow Cytometer (BD Biosciences, Milpitas, USA). Hybridomas (1 × 10^6^ cells/mL) were collected, washed and then subjected to FACS isolation as described above. The gating and parameter settings were conducted via the software BD FACSDiva (BD Biosciences, Milpitas, USA), and the MFIs of two subpopulations were analyzed using FlowJo. After culturing for 10–14 days, viable subclones were collected, expanded and seeded into 24‐well plates at a density of 5×10^4^ cells/mL. Following 3 days of culture, the supernatants of each subpopulation (high‐mGFP and low‐mGFP) were collected. Their antibody production levels were compared by examining the concentrations of secreted IgG molecules, as described above. Furthermore, the recognition specificity of these supernatants was determined with antigen‐coated (BFNB‐OVA, 0.5 mg/L) plates via non‐competitive ELISA.

### 4.19 Statistical Analysis

Each treatment was performed with three biological replicates. All data are presented as the mean ± standard deviation and were analyzed using Student's *t*‐test. Statistical analysis was carried out with Prism 8.0. Meanwhile, ^*^: *p*‐value < 0.05, ^**^: *p*‐value < 0.01, ^***^: *p*‐value < 0.001, ^****^: *p*‐value < 0.0001 were used to indicate statistical significance. The ns indicates not significant.

## Author Contributions

R.Z. and Y.G. contributed to the conceptualization of the study. The investigation was carried out by R.Z., X.L., Y.L., P.Y., and S.S., while the methodology was developed by R.Z. and X.L. Visualization was handled by R.Z., and data curation was performed by X.L. and Y.L. The project was supervised by Y.L., Y.M., G.Z., J.S.L., and Y.G. R.Z. prepared the original draft of the manuscript, and X.L., Y.M., J.S.L., and Y.G. contributed to reviewing and editing. Fundings for the project were acquired by R.Z., J.S.L., and Y.G.

## Conflicts of Interest

The authors declare that they have no competing interests.

## Supporting information




**Supporting File**: advs73817‐sup‐0001‐SuppMat.docx.

## Data Availability

The high‐throughput BCR‐Seq data underlying this article is available in NCBI Sequence Read Archive (SRA) database (httlp://ncbi.nlm.nih.gov/sra)with BioProject number of PRJNA1272265. The mass spectrometry proteomics data have been deposited to the ProteomeXchange Consortium (http://www.proteomexchange.org/) via the PRIDE partner repository with the dataset identifier PXD064735. Further source data and constructs will be shared on reasonable request to the corresponding author.
