# Supplementary material for: Quantitative Proteomics and CRISPR/Cas9 Editing Reveal UPR‐Mediated Control of Immunoglobulin Homeostasis in Hybridomas
Source: Adv Sci (Weinh). 2026 Jan 20;13(17):e14140. doi: 10.1002/advs.202514140 (PMC13042447; doi:10.1002/advs.202514140)
Supplement: Supplementary file 1 — Supporting File: advs73817‐sup‐0001‐SuppMat.docx. [file ADVS-13-e14140-s001.docx]

**Supplementary Materials**

**Quantitative Proteomics and CRISPR/Cas9 Editing Reveal UPR-Mediated Control of Immunoglobulin Homeostasis in Hybridomas**

Rubing Zou ^a†^, Xinying Lu ^a†^, Ying Liu ^a^, Peiyu Yang ^a^, Shuo Sun ^b^, Yihua Liu ^c^, Yinyuan Mo ^d^, Guonian Zhu ^a^, Jae Seong Lee ^e,f*^, Yirong Guo ^a*^

^a^ Institute of Pesticide and Environmental Toxicology, Zhejiang Key Laboratory of Biology and Ecological Regulation of Crop Pathogens and Insects, Ministry of Agriculture and Rural Affairs Key Laboratory of Molecular Biology of Crop Pathogens and Insect Pests, Zhejiang University, Hangzhou, 310058, China;

^b^ School of Life Science, Jining Medical University, Rizhao, 276826, China;

^c^ Research Institute of Subtropical Forestry, Chinese Academy of Forestry, Hangzhou, 311400, China;

^d^ Institute of Clinical Medicine, Zhejiang Provincial People’s Hospital of Hangzhou Medical College, Hangzhou, 310014, China;

^e^ Graduate School of Engineering Biology, KAIST, Daejeon, 34141, Republic of Korea.

^f^ KI for the BioCentury, KAIST, Daejeon, 34141 Republic of Korea;

^†^ These authors contributed equally to this work.

*Correspondence to: Jae Seong Lee (Email: jaeseonglee@kaist.ac.kr).

*Correspondence to: Yirong Guo. (Email: [yirongguo@zju.edu.cn](mailto:yirongguo@zju.edu.cn)).

**Figure S1 Characterization of hybridomas through a non-competitive ELISA (Dot) and IgG quantification (Bar)**


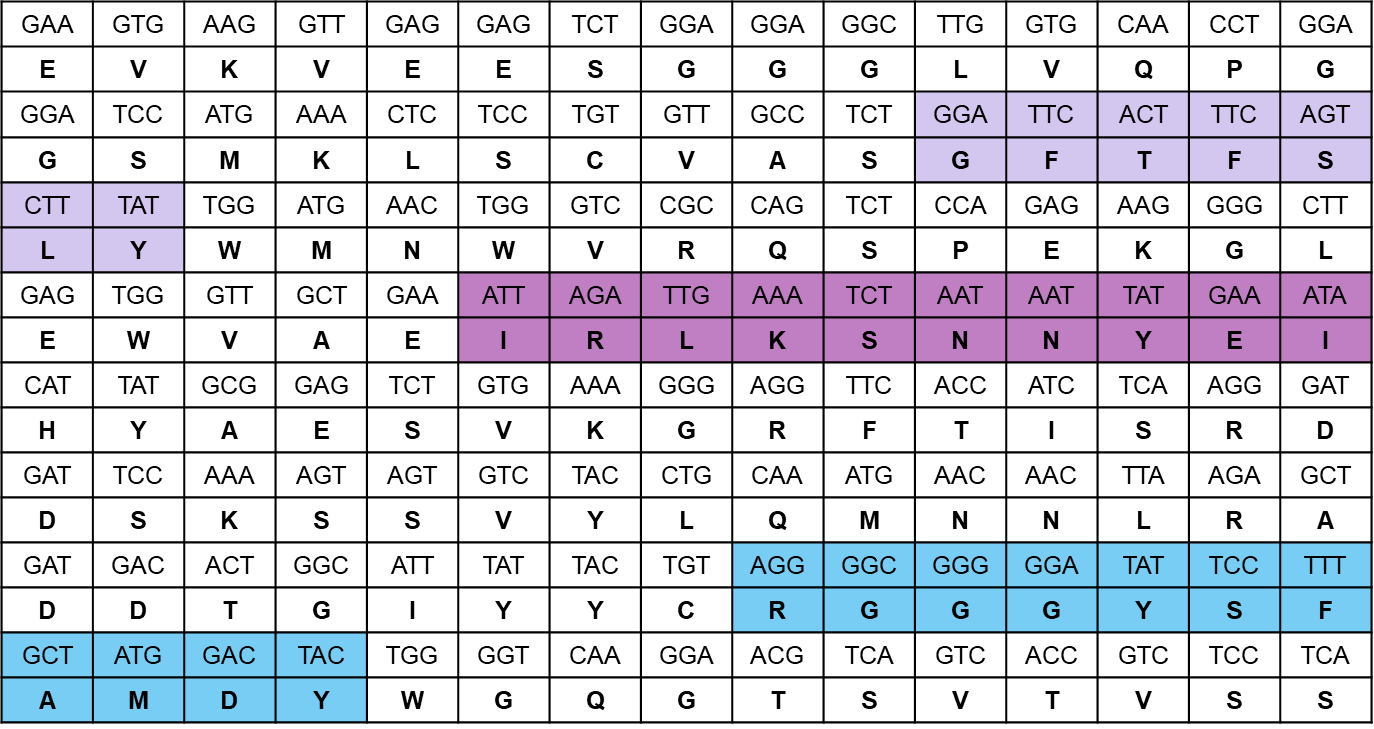


**Figure S2 The alignments of VH with the IgBLAST database.** The identified CDR1, CDR2 and CDR3 regions were marked in light purple, dark purple and blue, respectively.


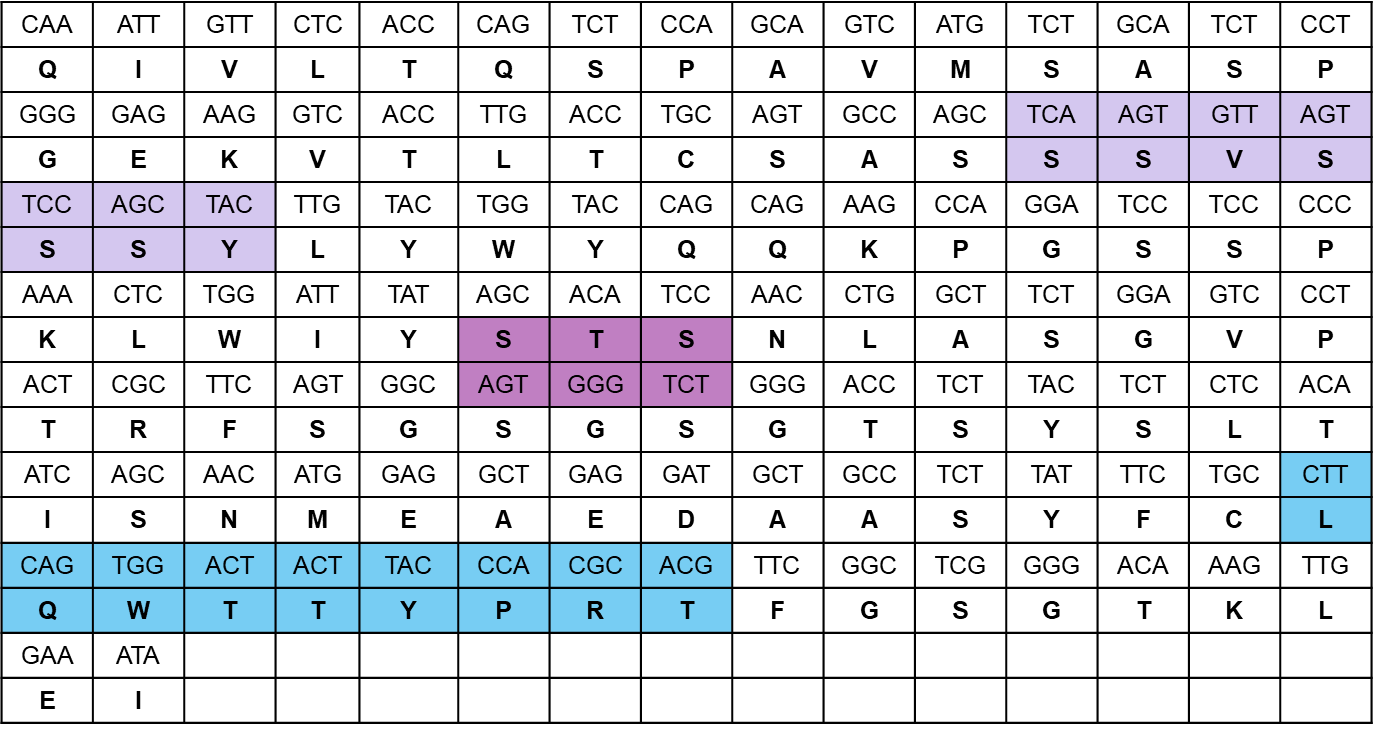
Figure S3 The alignments of VL with the IgBLAST database. The identified CDR1, CDR2 and CDR3 regions were marked in light purple, dark purple and blue, respectively.


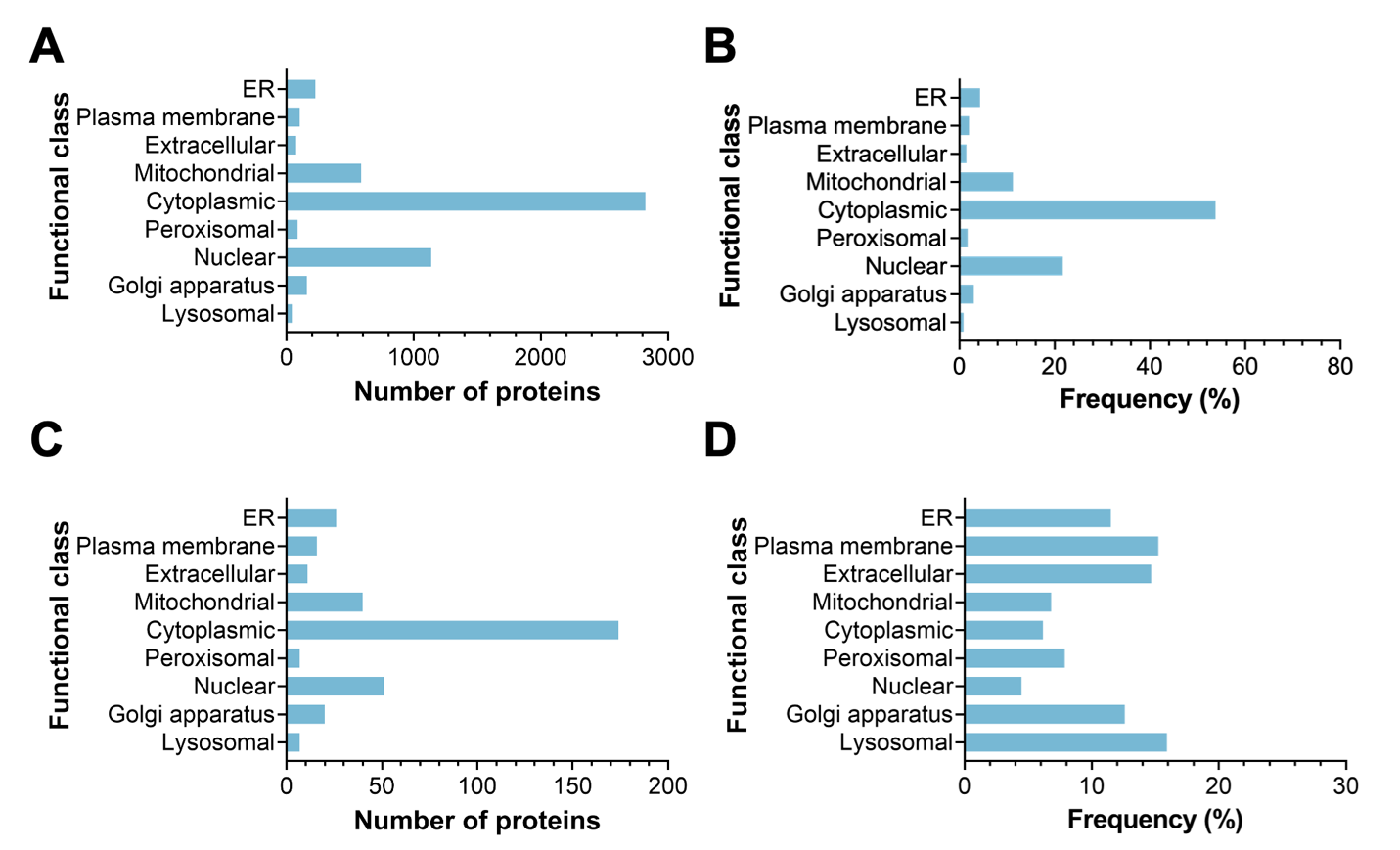


**Figure S4** **Sub-cellular localization annotation of proteins.** (**A-B**) Number (**A**) and frequency (**B**) of total expressed proteins. (**C-D**) Number (**C**) and frequency (**D**) of differentially expressed proteins (DEPs).

**Figure S5 ER-Tracker staining results of functional and non-functional hybridoma cells.** (**A**) Fluorescence intensity of BJQ-E2 and BJQ-D4. (**B**) Fluorescence intensity of BFNB-4E and BFNB- 11F. *** and **** indicated *p* < 0.001 and *p* < 0.0001, respectively.


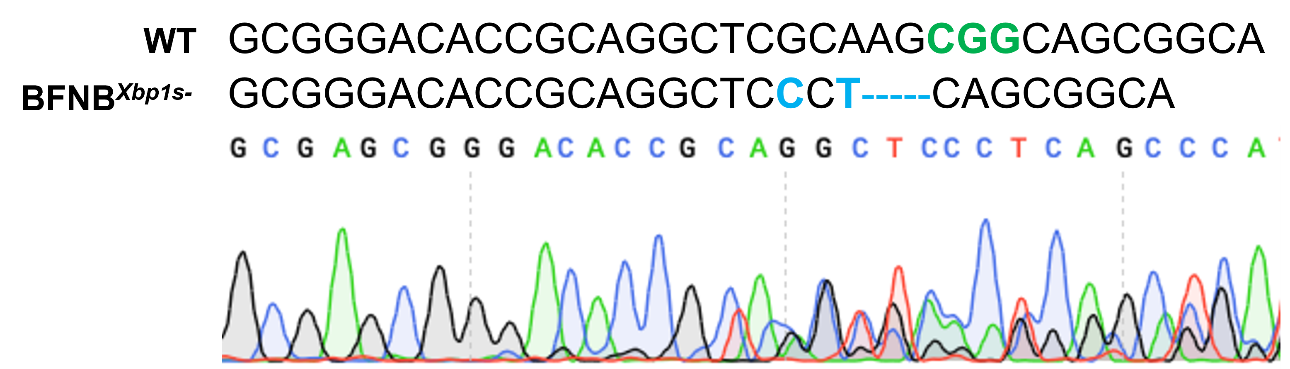


**Figure S6 PCR amplification of *Xbp1s* in** **BFNB*^Xbp1s-^* knockout cell line**


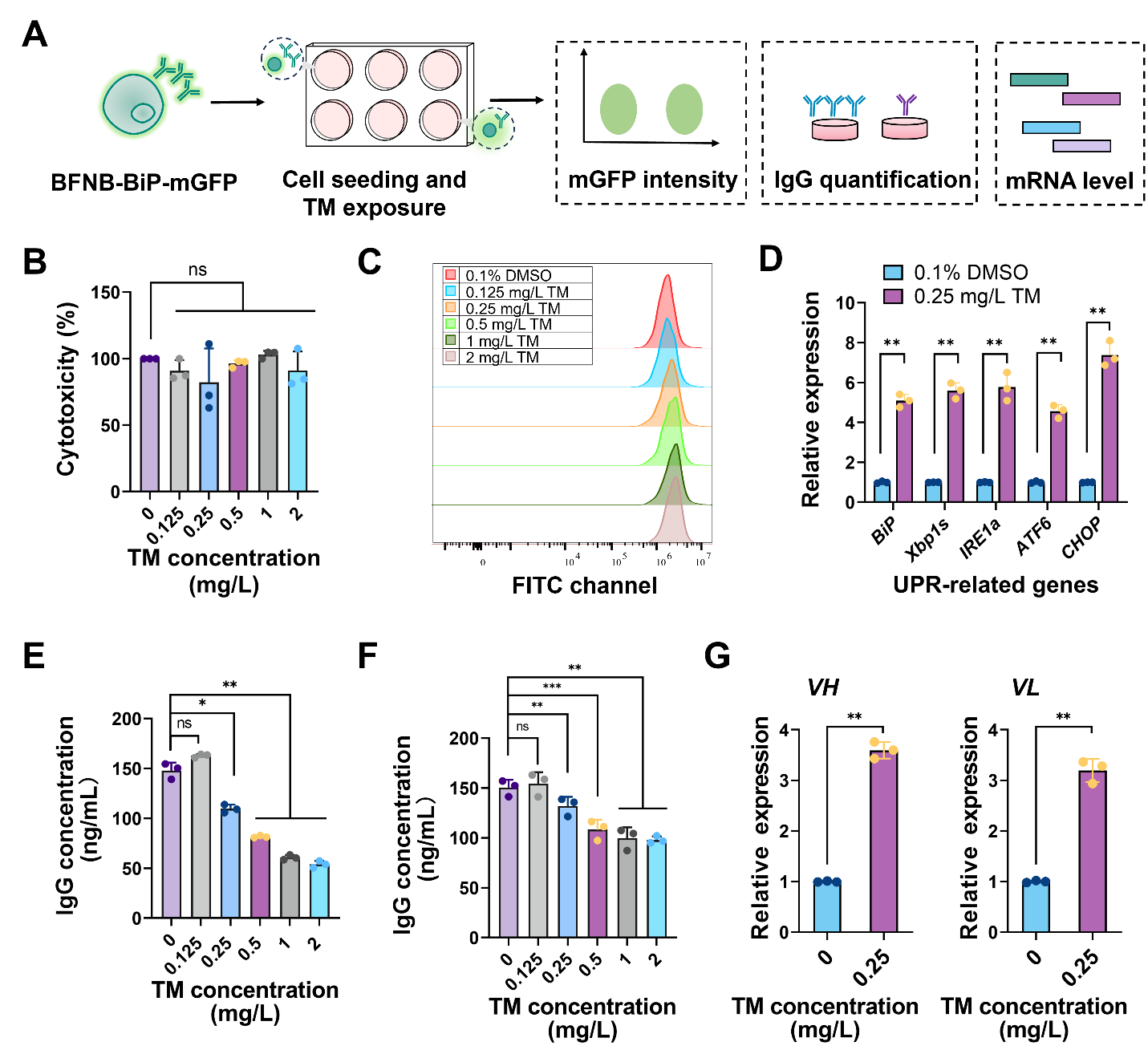


**Figure S7 Investigation of tunicamycin (TM) exposure on UPR activation and antibody production in hybridomas.** (**A**) Schematic illustration. The correlation between UPR activation and antibody production was evaluated in the engineered BFNB-BiP-mGFP cell line by measuring intracellular mGFP signal, secreted IgG amount and antibody mRNA expression following induction with TM, a well-known ER stress inducer. (**B**) Cytotoxicity assessment of TM exposure in BFNB-BiP-mGFP using a CCK8 kit. (**C**) Intracellular mGFP intensity in BFNB-BiP-mGFP cells treated with varying TM concentrations (0.125~2 mg/L, 0.1% DMSO). (**D**) Effects of TM exposure (0.25 mg/L) on the mRNA expression of UPR-related genes in BFNB-BiP-mGFP cells. Cells treated with 0.1% DMSO served as the negative control. (**E-F**) Secreted IgG levels in BFNB-BiP-mGFP (**F**) and WT (**F**) cells under varying TM concentrations. (**G**) Effects of TM exposure (0.25 mg/L) on the mRNA expression of antibody genes in BFNB-BiP-mGFP cells. Cells treated with 0.1% DMSO served as the negative control. Three biological replicates were performed and error bars represent the standard deviation. Statistical significance was analyzed using Student’s t-test, while ns, *, ** and *** represent not significant, *p*-value of < 0.05, < 0.01 and < 0.001, respectively.


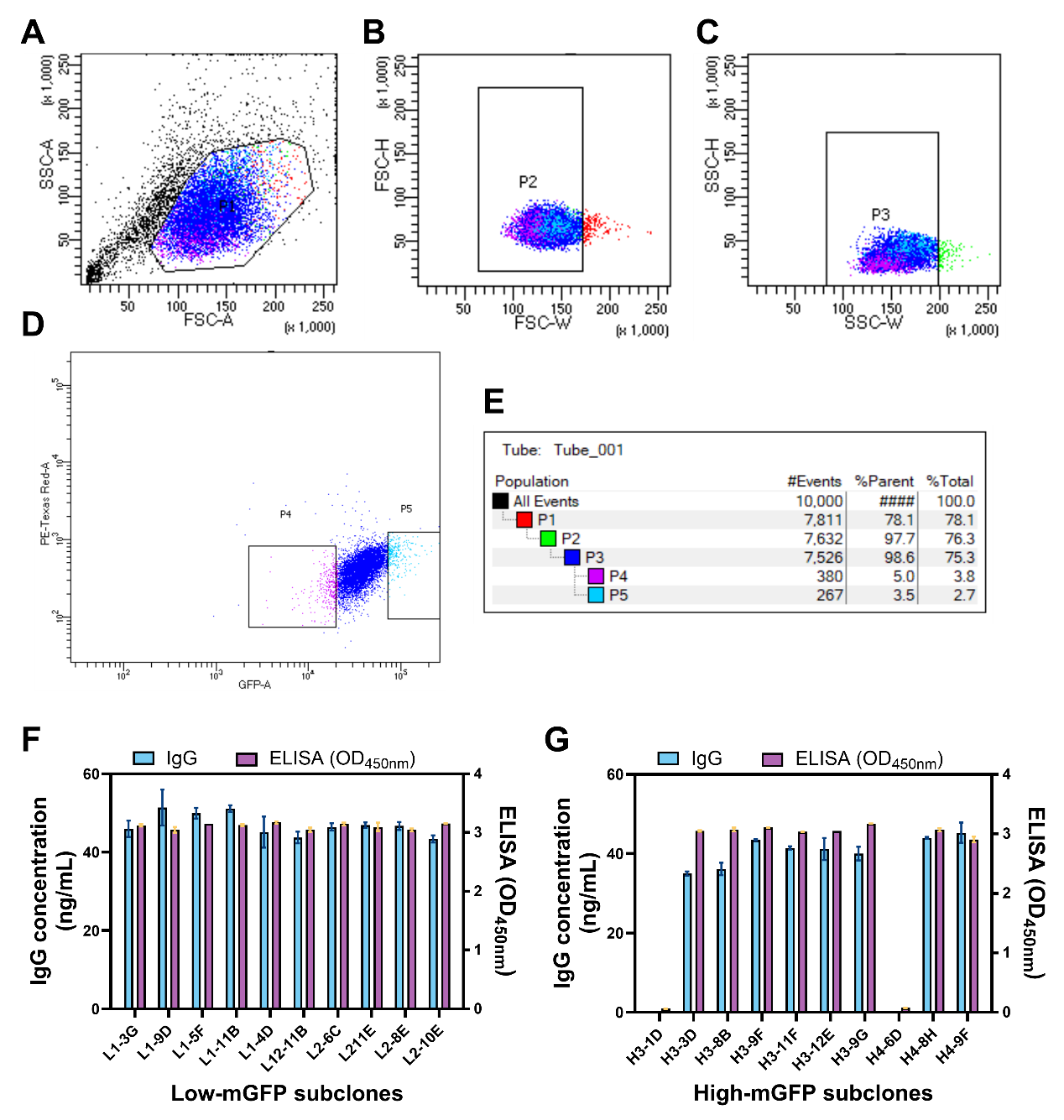


**Figure S8** **Isolation of BFNB-BiP-mGFP subclones with varying mGFP intensity through fluorescence-activated cell sorting (FACS).** (**A**) Forward scatter (FSC) and side scatter (SSC) plot. (**B**) FSC-W and FSC-H plot. (**C**) SSC-W and SSC-H plot. (**D**) Gating strategy to identify high-mGFP and low-mGFP subpopulations. (**E**) Distribution of the identified subpopulations. (**F-G**) Secreted IgG levels and binding absorbance (OD_450nm_) for ten isolated subclones from the subpopulations of low-mGFP (n=10) (**F**), and ten isolated subclones from the subpopulations of high-mGFP (n=10) (**G**). The x-axis shows the name of each subclone. The binding ability was examined via non-competitive ELISA plates that coated with BFNB-OVA (0.5 mg/L).

**Table S1 Identification of germline genes of VH and VL through IgBLAST**

| **Chain** | **V region (similarity, %)** | **J region (similarity, %)** | **V-J frame** | **Productive** |
| --- | --- | --- | --- | --- |
| VH | *Ighv*-6-6*02, 96.9% | *Ighj4**01, 94.3% | In-frame | Yes |
| VL | *Igκv*4-79*01,97.2% | *Igκj4**01, 100% | In-frame | Yes |

**Table S2 The profiled V/J pair and CDR3 region of VH in BJQ-E2 and BJQ-D4 via BCR-Seq**

| **Seq No.** | **BJQ-E2** | | | **BJQ-D4** | | |
| --- | --- | --- | --- | --- | --- | --- |
|  | **V/J pair** | **CDR3 amino acid** | **Clone fraction (%)** | **V/J pair** | **CDR3** | **Clone fraction (%)** |
| VH-1 | *Ighv6-6*/*Ighj4* | CRGGGYSFAMDYW | 99.81 | *Ighv6-6*/*Ighj4* | CRGGGYSFAMDYW | 99.99 |
| VH-2 | *Ighv6-3/Ighj4* | CRGGGYSFAMDYW | 0.13 | *Ighv6-6*/*Ighj4* | CRGGGYSFAGDGLAGP | 0.005 |
| VH-3 | *Ighv1-4*/*Ighj2* | CERRDWEGDYW | 0.03 | *Ighv6-6*/*Ighj3* | CRDWFAYW | 0.005 |
| VH-4 | *Ighv1-15*/*Ighj1* | CTRERDYYGTWYFDVW | 0.01 |  |  |  |
| VH-5 | *Ighv1-64*/*Ighj2* | CARWELVDYW | 0.01 |  |  |  |
| VH-6 | *Ighv5-9*/*Ighj4* | CNAPEVLTTDWRRGNYW | 0.01 |  |  |  |
| VH-7 | *Ighv1-83*/*Ighj1* | CARYFYDYDRGYFDVW | 0.01 |  |  |  |

**Table S3 The profiled V/J pair and CDR3 region of VL in BJQ-E2 and BJQ-D4 via BCR-Seq**

| **Seq No.** | **BJQ-E2** | | | **BJQ-D4** | | |
| --- | --- | --- | --- | --- | --- | --- |
|  | **V/J pair** | **CDR3 amino acid** | **Clone fraction (%)** | **V/J pair** | **CDR3** | **Clone fraction (%)** |
| VL-1 | *Igκv4-79*/*Igκj4* | CLQWTTYPRTF | 99.98 | *Igκv4-79*/*Igκj4* | CLQWTTYPRTF | 99.99 |
| VH-2 | *Igκv6-25/Igκj1* | CQQHYSTPPTF | 0.004 | *Igκv4-79*/*Igκj4* | CLQWTYPRTF | 0.005 |
| VH-3 | *Igκv6-17*/*Igκj5* | CQQHYNTPLTF | 0.004 |  |  |  |
| VH-4 | *Igκc1-110*/*Igκj2* | CSQSTHVPYTF | 0.004 |  |  |  |

**Table S4 Parameters for Maxquant analysis.**

| **Item** | **Value** |
| --- | --- |
| Maxquant version | 2.0.3.1 |
| Protein Database | Mus_musculus.GRCm38.pep.all.fa_unique.fasta |
| Cys alkylation | Iodoacetamide |
| Dynamic Modification | Oxidation (M), Acetyl (Protein N-Terminus), |
| Static Modification | Carbamidomethyl (C) |
| Enzyme Name | Trypsin/P |
| Max. Missed Cleavage Sites | 2 |
| Precursor Mass Tolorance | 10 ppm |

**Table S5 Primers for validation of knockout and knock-in through CRISPR/Cas9**

| **Target** | **Forward (5’-3’)** | **Reverse (5’-3’)** |
| --- | --- | --- |
| Xbp1s | CCAAAGTGCTACTCTTATCTGGCC | TCGGGAGCTCCTGCACACA |
| BiP-mGFP | AGGATGTGATCTGGGTTTCACA | GGGCTTGGACTAAACACAGC |

**Table S6 Primers for UPR-related genes and antibody genes via qRT-PCR**

| **Target** | **Forward (5’-3’)** | **Reverse (5’-3’)** |
| --- | --- | --- |
| *Xbp1s* | AAGAACACGCTTGGGAATGG | CTGCACCTGCTGCGGAC |
| *BiP* | TCATCGGACGCACTTGGAA | CAACCACCTTGAATGGCAAGA |
| *IRE1a* | CTGGCTTCTCATAGGACACCAT | TCTCGATGTTTGGGCAGGTT |
| *ATF6* | GGAGAGGTGTCTGTTTCGGG | AAACAACGTCGACTCCCAGT |
| *CHOP* | GTCCCTAGCTTGGCTGACAGA | TGGAGAGCGAGGGCTTTG |
| *VH* | GGCTGGATTCGTCAGTCTCC | TGACCACACTGGCGATCTTG |
| *VL* | GTGCTGACCCAAACTCCACT | CTCTGCAGGGACCAGTGAAAA |
| *Ighg1* | CCCAAGGATGTGCTCACCAT | CCTGGTGCATGATGGGAAGT |
| *Igκc* | GCGTCCTGAACAGTTGGACT | TCGTCCTTGGTCAACGTGAG |
| *GADPH* | GGTGAAGGTCGGTGTGAACG | CTCGCTCCTGGAAGATGGTG |
